# Susceptibility to stress and nature exposure: Unveiling differential susceptibility to physical environments; a randomized controlled trial

**DOI:** 10.1371/journal.pone.0301473

**Published:** 2024-04-17

**Authors:** Aaron M. Eisen, Gregory N. Bratman, Hector A. Olvera-Alvarez

**Affiliations:** 1 School of Nursing, Oregon Health & Science University, Portland, OR, United States of America; 2 School of Environmental and Forest Sciences, University of Washington, Seattle, WA, United States of America; 3 Department of Psychology, University of Washington, Seattle, WA, United States of America; 4 Department of Environmental and Occupational Health Sciences, University of Washington, Seattle, WA, United States of America; University of Rome La Sapienza: Universita degli Studi di Roma La Sapienza, ITALY

## Abstract

**Background:**

Emerging epidemiological evidence indicates nature exposure could be associated with greater health benefits among groups in lower versus higher socioeconomic positions. One possible mechanism underpinning this evidence is described by our framework: (susceptibility) adults in low socioeconomic positions face higher exposure to persistent psychosocial stressors in early life, inducing a pro-inflammatory phenotype as a lifelong susceptibility to stress; (differential susceptibility) susceptible adults are more sensitive to the health risks of adverse (stress-promoting) environments, but also to the health benefits of protective (stress-buffering) environments.

**Objective:**

Experimental investigation of a pro-inflammatory phenotype as a mechanism facilitating greater stress recovery from nature exposure.

**Methods:**

We determined differences in stress recovery (via heart rate variability) caused by exposure to a nature or office virtual reality environment (10 min) after an acute stressor among 64 healthy college-age males with varying levels of susceptibility (socioeconomic status, early life stress, and a pro-inflammatory state [inflammatory reactivity and glucocorticoid resistance to an in vitro bacterial challenge]).

**Results:**

Findings for inflammatory reactivity and glucocorticoid resistance were modest but consistently trended towards better recovery in the nature condition. Differences in recovery were not observed for socioeconomic status or early life stress.

**Discussion:**

Among healthy college-age males, we observed expected trends according to their differential susceptibility when assessed as inflammatory reactivity and glucocorticoid resistance, suggesting these biological correlates of susceptibility could be more proximal indicators than self-reported assessments of socioeconomic status and early life stress. If future research in more diverse populations aligns with these trends, this could support an alternative conceptualization of susceptibility as increased environmental sensitivity, reflecting heightened responses to adverse, but also protective environments. With this knowledge, future investigators could examine how individual differences in environmental sensitivity could provide an opportunity for those who are the most susceptible to experience the greatest health benefits from nature exposure.

## Introduction

Emerging epidemiological evidence indicates that nature (e.g., green spaces) exposure could be associated with better health among individuals in low socioeconomic positions to a greater degree than among more privileged groups [1–4]. This implies that nature exposure within urban settings could potentially attenuate the adverse effects of chronic stress on health with the greatest impact among individuals who are the most susceptible to life stressors [5–7]. However, the evidence supporting this possibility primarily comes from cross-sectional and observational studies, including reports of null associations [8–10]. Further investigation is necessary to better understand this phenomenon, especially through experimental paradigms that can provide insight into potential causal mechanisms. Establishing experimental evidence could further support the notion that nature-based interventions (e.g., increasing access to urban parks) could help curb health disparities across socioeconomic conditions.

One possible mechanism underpinning this phenomenon is outlined by integrating three overarching ideas: (a) individuals in low socioeconomic conditions often experience higher exposure to persistent psychosocial stressors in early life, inducing a lifelong susceptibility to stress; (b) nature exposure protects against the adverse health effects of stress; (c) susceptibility to stress could reflect increased sensitivity to environmental conditions, inducing greater health benefits from the protective effects of nature exposure among individuals in lower versus higher socioeconomic positions.

### Early life stress

Evidence demonstrates that individuals in low socioeconomic positions, compared to those in higher positions, often experience higher exposure to persistent psychosocial stressors in early life [11–15]. Evidence also indicates that early life stress can produce a lifelong susceptibility to stress through various allostatic pathways (Biological Embedding Model [16–26]). In this context, one well-established neurobiological pathway consists of early life stress inducing a pro-inflammatory phenotype, observed as increased neuro-immune reactivity to psychological and biological stressors combined with resistance to anti-inflammatory signals (Neuro-Immune Network Hypothesis [27, 28]).

In short, when the amygdala activates in response to perceived threats, the sympathetic-adrenal-medullary axis engages within seconds to trigger the sympathetic nervous system, sending a cascade of messengers (e.g., catecholamines) that are received by monocytes as pro-inflammatory signals [29]. Minutes later, the hypothalamic-pituitary-adrenal axis engages, sending another cascade of messengers (e.g., glucocorticoids) that are received by monocytes as anti-inflammatory signals [29]. Importantly, early life stress has been shown to augment the reactivity of monocytes to these signals (heightened sensitivity to pro-inflammatory signals, but also, decreased sensitivity to anti-inflammatory signals), leading to a pro-inflammatory immune state, which has been shown to persist throughout adulthood (“brain to immune traffic” [27, 28]). To this end, inflammation is systemic and induces neuro-inflammation which has also been shown to elevate amygdala reactivity to perceived threats (“immune to brain traffic” [27, 28]) in a self-sustaining cycle.

As an example of this pro-inflammatory phenotype, experimental evidence has shown that early life stress is associated with increased monocyte production of pro-inflammatory cytokines (e.g., interleukin-6; IL-6) among healthy adults in response to psychosocial stressors [30, 31]. Other evidence has shown that early life stress is associated with increased monocyte production of pro-inflammatory cytokines (e.g., IL-6) and resistance to glucocorticoids (e.g., cortisol) among healthy adults in response to in vitro bacterial challenges [32–34]. Importantly, evidence also indicates that higher socioeconomic status during adulthood is unable to reverse these alterations [32, 35].

Together, these findings suggest that early life stress becomes biologically embedded through a pro-inflammatory phenotype that, when combined with persistent exposure to stressors, could result in chronic inflammation and consequently heighten the risk of developing diseases of aging [27, 28]. This risk is particularly elevated among adults in low socioeconomic positions, who are more likely to experience early life stress, current life stressors, and health disparities across these diseases [32, 35]. In this context, a pro-inflammatory phenotype could represent a more sensitive and relevant indicator as a biological correlate of susceptibility to stress (proximal; shorter pathway between indicators and outcomes), compared to self-report assessments of socioeconomic status and early life stress (distal; longer pathway between indicators and outcomes that in turn, increases the risk of unmeasured confounders).

### Nature exposure

Psychoevolutionary theories posit that many types of nature exposure are health-protective relative to exposure to urban settings, as human beings share an innate physiological affinity to natural features that afforded safety and nourishment throughout our evolutionary history [36–39]. Due to these connections, nature exposure can have a positive effect on health through stress-related mechanisms, including better stress recovery (Stress Reduction Theory [39–41]). This notion is supported by experimental evidence of increased parasympathetic and reduced sympathetic activation within natural versus urban settings following sympathetic arousal induced by an acute psychosocial stressor [41–46].

For instance, a landmark study found increased parasympathetic (pulse transit time) and reduced sympathetic activation (muscle tension and skin conductance) during recovery from heightened sympathetic arousal induced by an acute stressor (video depicting serious injuries) among healthy adults exposed to videos of natural versus urban environments [41]. More recent studies have also reported similar findings, showing increased parasympathetic and reduced sympathetic activation (e.g., heart rate variability) during recovery from sympathetic arousal induced by an acute stressor (Trier Social Stress Test; TSST) among healthy adults exposed to natural environments through virtual reality, compared to an indoor office environment [45, 46].

Therefore, better stress recovery (increased parasympathetic and reduced sympathetic activation after an acute stressor) is one plausible mechanism underpinning the health-protective effects of nature [41, 47, 48]. More specifically, evidence supports the notion that incorporating nature into residential settings could buffer against the effects of chronic stress [49–51], reducing the risk of various stress-induced health outcomes [52–55] as demonstrated by the epidemiological evidence [1–4]. In this investigation, we centered our interpretations on cardiovascular disease per the direct and observable link between autonomic and cardiovascular functioning, evidenced by changes in heart rate and blood pressure.

### Differential susceptibility

We propose that adults living in lower versus higher socioeconomic positions could experience greater cardiovascular benefits from nature exposure through increased sensitivity to environmental conditions as a function of susceptibility to stress. In this context, a pro-inflammatory phenotype induced by early life stress could heighten the effects of various environmental conditions; although research has primarily centered on the negative effects of adverse (stress-promoting) environments, growing evidence indicates a link to better outcomes within protective (stress-buffering) environments. This notion is grounded within neurodevelopmental theories [56–59] which converge on the Differential Susceptibility Hypothesis [59], positing that susceptible individuals, compared to less susceptible individuals, are more sensitive to adverse, but also protective environmental conditions.

In support of this hypothesis, a review of fifty-six studies, encompassing thousands of participants (*n* = ~ 23K) consistently noted that individuals who were susceptible to stress (using a broad range of phenotypic, endophenotypic, and genotypic indicators) showed greater physical and mental health benefits within protective social conditions (e.g., positive feedback, social support) relative to less susceptible participants [60]. Emerging evidence also indicates differential susceptibility could be relevant for the specific susceptibility indicators employed in this investigation, including socioeconomic status, early life stress, and a pro-inflammatory phenotype.

For instance, regarding socioeconomic status, a landmark study on the population of England below the age of retirement (*n* = ~ 41M) found that the cardiovascular disparity gap between the highest and lowest income groups was 30% smaller in neighborhoods with the highest versus lowest levels of nature exposure [1]. Regarding early life stress, a national longitudinal survey of adults in the United States (*n* = ~ 34K) provided evidence that adults with adverse childhood experiences showed greater decreases in transdiagnostic psychopathological factors following annual reductions in current life stress, compared to adults without adverse childhood experiences [61]. Regarding a pro-inflammatory phenotype, experimental evidence has shown that healthy adults (*n* = 61) exposed to an in vivo inflammatory challenge (low-dose endotoxin) demonstrated greater neural activity in reward processing regions (ventral striatum and ventromedial prefrontal cortex) when receiving positive versus neutral social feedback on a pre-recorded mock job interview, compared to adults who were given a placebo (*n* = 57 [62]).

Throughout this body of evidence, a trend emerges in which susceptible individuals exhibit better outcomes in protective environments compared to less susceptible individuals. In essence, susceptible individuals tend to exhibit greater sensitivity to both adverse and protective environmental influences than their less susceptible counterparts, who appear to exhibit relatively moderate effects from their environment [59].

### Current investigation

**Fig 1:**
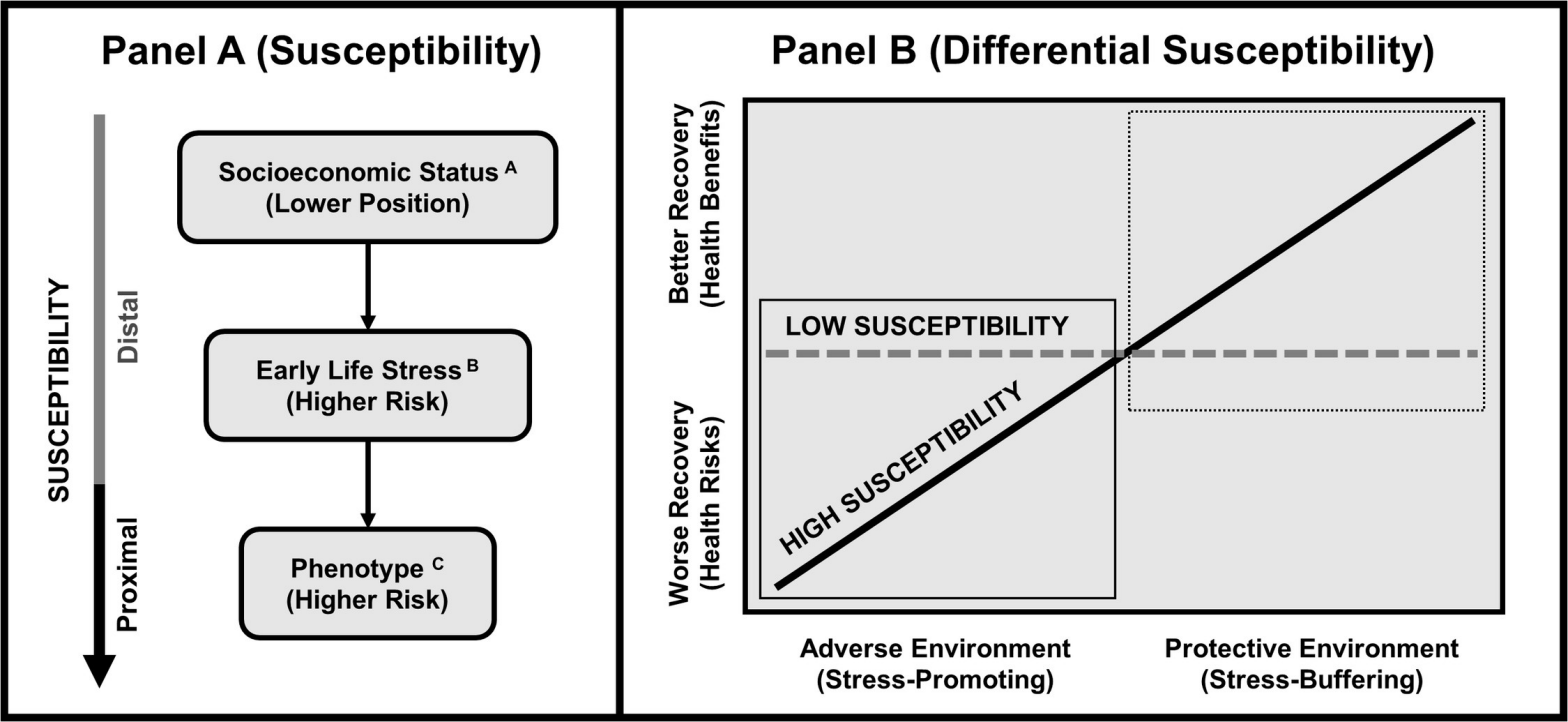
Theoretical framework. Emerging epidemiological evidence indicates that nature exposure could be associated with cardiovascular health among individuals in low socioeconomic positions to a greater degree than among more privileged groups. One possible mechanism underpinning this evidence is described by our theoretical framework: (Panel A; Susceptibility [Measures]) individuals in low socioeconomic positions^A^, compared to those in higher positions, are at risk for higher exposure to stressors in early life^B^ and a pro-inflammatory phenotype^C^ induced by early life stress; (Panel B; Differential Susceptibility [Outcomes]) susceptible individuals are more sensitive to the health risks of adverse environments (e.g., worse recovery from stress), relative to less susceptible individuals, but also are more sensitive to the health benefits of protective environments (e.g., better recovery from stress), relative to less susceptible individuals. (A) Socioeconomic status is defined as the position of an individual in their society which is determined by both social and economic factors that impact exposure to and experiences with psychosocial stressors [63]. (B) Early life stress is defined as persistent exposure to stressors during childhood, with ranging degrees of perceived severity, that induce psychobiological responses and could promote neurodevelopmental alterations over time [64]. (C) A pro-inflammatory phenotype is defined as increased neuro-immune reactivity to psychological and biological stressors combined with resistance to anti-inflammatory signals [27, 28]. Panel B was adapted from “For Better and For Worse: Differential Susceptibility to Environmental Influences” by Jay Belsky, Marian J. Bakermans-Kranenburg, and Marinus H. van IJzendoorn, 2007, Current Directions in Psychological Science, 16(6), 300–304. Copyright 2007 by Association for Psychological Science. Adapted with permission.

Embedding this theory into our framework (Fig 1) implies that adults in lower versus higher socioeconomic positions (who are at risk for higher exposure to stressors in early life [11–15] and a pro-inflammatory phenotype induced by early life stress [27, 28]), are more likely to exhibit greater cardiovascular risks in adverse environments, but also greater cardiovascular benefits in protective environments [59]. Concordantly, we expect that adults in lower versus higher socioeconomic positions are more likely to experience: (a) early life stress; (b) a pro-inflammatory phenotype induced by early life stress; (c) increased sensitivity to the cardiovascular benefits of natural environments (increased parasympathetic and reduced sympathetic activation [41, 45, 46]) as a function of differential susceptibility. Considering that evidence indicates a pro-inflammatory phenotype is a key mechanism driving the association between early life stress and negative outcomes under adverse conditions [27, 28], we expect that it could also be a key mechanism leading to positive outcomes under protective conditions.

The aforementioned evidence supports the possibility that adults with a pro-inflammatory phenotype could experience greater cardiovascular benefits from the stress-buffering effects of nature. However, to our knowledge, this mechanism has never been explored in an experimental paradigm. The purpose of this investigation was to: (a) posit a theoretical framework to highlight potential mechanisms underpinning differential susceptibility to natural environments, and (b) provide initial insight on the hypothesized associations to determine if there is sufficient evidence that supports more comprehensive investigations in larger and more diverse samples.

In this controlled experimental study, we investigated stress recovery (autonomic activation) following exposure to an acute psychosocial stressor (TSST) and subsequently a nature (public park) or comparator (indoor office) environment among participants with varying levels of susceptibility. We hypothesized that participants with higher susceptibility to stress would demonstrate greater stress recovery (increased parasympathetic and reduced sympathetic activation) when exposed to a virtual nature environment, following the acute stressor.

The environmental exposures (nature and office) were conducted using a virtual reality paradigm as immersive modalities have been shown to induce responses comparable to real world exposures [65, 66] and offer several key experimental advantages. These advantages include the mitigation of confounding factors (e.g., exercise, air pollution, noise, temperature, humidity, light [52]), isolation of sensory effects (e.g., visual, auditory [65]), and greater control over the exposure (e.g., duration, proximity, spatial scale, presence of humans or animals [66]).

Stress recovery was operationalized by indicators of autonomic activation (heart rate variability [67]), that were expressed as sympathetic and parasympathetic profiles. Autonomic activation was used to index stress recovery as the homeostasis of this nervous system indicates neuro-cardiac interactions in response to various environmental conditions [68, 69], responds rapidly to acute exposures [69, 70], and is associated with cardiovascular health [67–74].

We also tested distal and proximal indicators of susceptibility in the context of our theoretical framework (Fig 1). Distal indicators included socioeconomic status (subjective social status) and early life stress (adverse childhood experiences); proximal indicators included two immunological assessments of monocytes to index a pro-inflammatory phenotype: (a) higher inflammatory reactivity to an in vitro bacterial challenge, and (b) higher glucocorticoid resistance during the inflammatory response to the in vitro bacterial challenge.

## Materials and methods

### Participants

We recruited male students (*n* = 64), 18–30 years of age, from a Texas university using local advertisements between April 1^st^ and July 31^st^, 2019. Participants were fluent English speakers, non-smokers, non-drug users, not diagnosed with or taking medication for chronic illness (diabetes, cardiovascular disease, metabolic syndrome, epilepsy, seizures, and asthma), and without a history of sleep problems; who were not night-shift workers and were naïve to our stressor. We recruited young adults to focus this stage of research on a healthier sample, as with age comes the development of aging-related health states that might confound the analyses. We also recruited exclusively young males (sex assigned at birth) to minimize variability in our outcome (stress recovery) that was not attributed to the experimental conditions or susceptibility indicators [75]. This was based on prior research showing that males demonstrate less variability than females in response to the same psychosocial stressor that was used in this investigation [76–78]. For instance, the literature supporting this stressor has provided evidence that hormonal fluctuations associated with menstrual cycles (e.g., estradiol, progesterone) and altered diurnal neuroendocrine rhythms associated with oral contraceptive use increase the variability of stress responses among females [79]. All participants provided written informed consent and were compensated with 40 USD for completing the experiment. This study was approved by the Institutional Review Board at the University of Texas at El Paso (1385515–3).

### Study design

**Fig 2:**
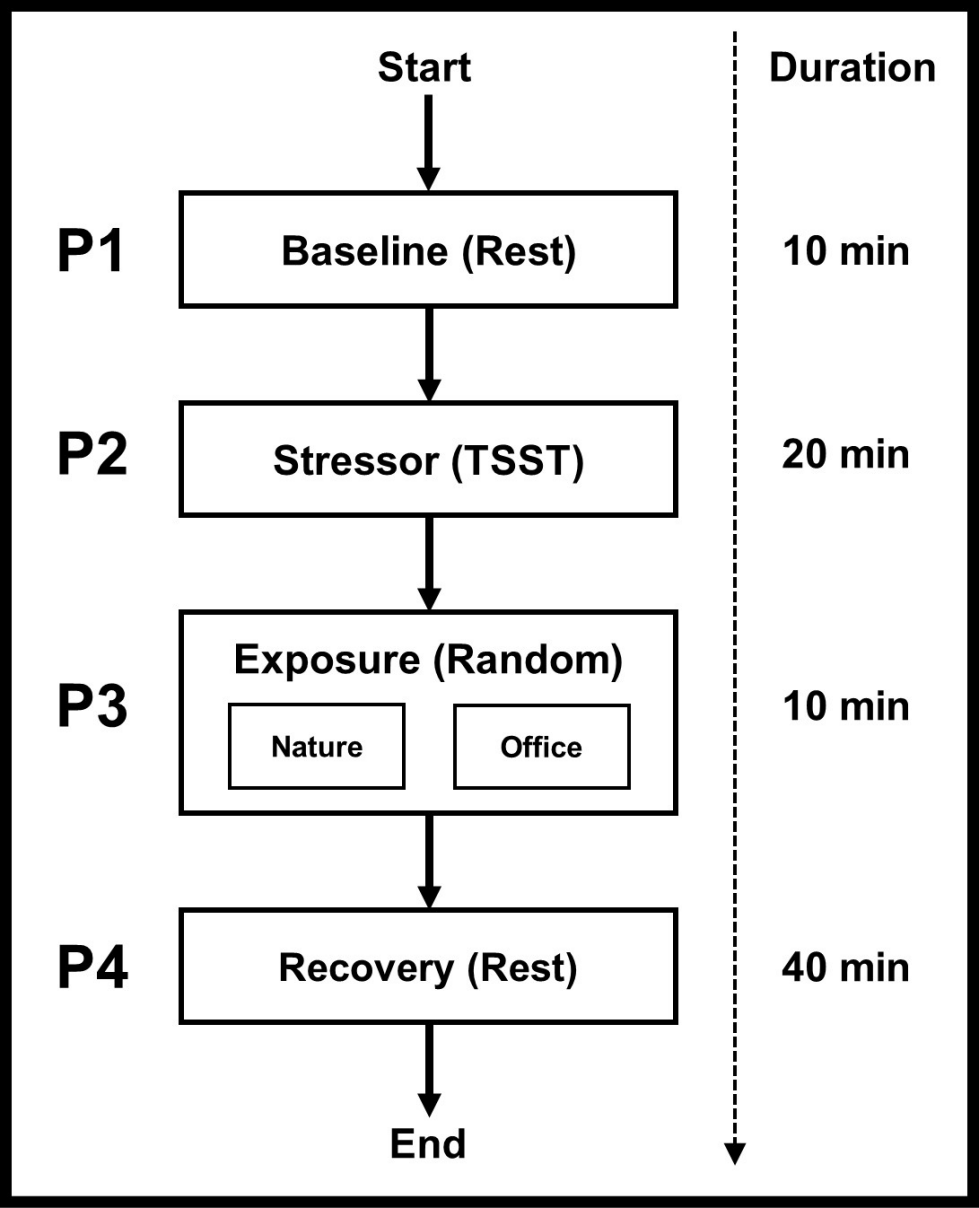
Flow diagram of exposure experiment. Stress recovery was assessed using heart rate variability to index changes in autonomic activation (sympathetic and parasympathetic) from baseline to the recovery period. Susceptibility indicators (socioeconomic status, early life stress, inflammatory reactivity, and glucocorticoid resistance) were assessed at various time points. Socioeconomic status (MacArthur Scale of Subjective Social Status) and early life stress (Adverse Childhood Experience Questionnaire) were measured during an initial visit to the laboratory within three days of the exposure experiment. Inflammatory reactivity and glucocorticoid resistance (in vitro bacterial challenge) were assessed using serum collected before the start of the exposure experiment.

We used a between-group design to compare the effects of nature versus indoor office environments on stress recovery from an acute psychosocial stressor, with a focus on the modifying effects of susceptibility to stress. We did this by having participants experience the psychosocial stressor, followed by a 10-minute exposure to a randomly assigned environment (nature or office) delivered using virtual reality, and subsequently a 40-minute recovery period. Changes in stress recovery were observed across the experiment (see Fig 2) and indexed using autonomic activation (heart rate variability). We then examined changes in autonomic activation as a function of the interaction between the environmental conditions and susceptibility to stress, represented as both distal indicators (socioeconomic status and early life stress) and proximal indicators (inflammatory reactivity and glucocorticoid resistance).

### Procedure

During an initial visit to the laboratory, participants underwent a health screening to determine the following exclusion criteria: body mass index ≥25 kg/m2, waist circumference ≥40 inches, blood pressure ≥140/90 mmHg, fasting glucose ≥100 mg/dL, and moderate or worse depressive severity (PHQ9 score ≥10 [80]). Participants then completed self-report questionnaires as measures of socioeconomic status and early life stress. Finally, all participants were exposed to a 5-minute interactive 360° VR video of Zion National Park, minimizing the potential for a “novelty” effect during the exposure experiment for those who had never experienced virtual reality [81]. Participants who passed the health screening were scheduled for the exposure experiment (Fig 2) on a separate day (within three days of the first visit) at a standardized time (9:00 am or 2:00 pm) to mitigate circadian effects [82].

#### Baseline

Upon arrival at the laboratory on the day of the exposure experiment, participants rested in an examination room (15 min) and then provided a blood sample (to perform bioassays of inflammatory reactivity and glucocorticoid resistance in response to an in vitro bacterial challenge). Subsequently, participants attached three wireless electrocardiogram leads to their abdomen and below their collarbone and reclined (supine) on an examination table (10 min) for the baseline assessment. Electrodes were connected wirelessly to a computer and remained on the participant for the duration of the experiment.

#### Stressor

Participants were taken to an office space devoid of natural elements to experience a variation of the TSST which has been shown to effectively simulate stressful situations in the real world [76, 83, 84]. The stressor exposure was coordinated by a female experimenter and performed in front of a male judge with two tasks, a public speaking task (why you would be the best candidate for your “dream job” during a mock job interview) and a mental arithmetic task (rapid and accurate serial subtraction). To increase the efficacy of the stressor, participants were informed that video monitoring was being conducted for the speech task and misinformed that the participant with the highest score on the mental arithmetic task would receive an additional incentive worth 30 USD. When participants stopped before the time allocated for the speech task, they were prompted to continue, with 10-second pauses on subsequent prompts. For the mental arithmetic task, when participants answered incorrectly, a buzzer was sounded, and they were prompted to restart from the beginning of the task. The stressor lasted for 20 min with four 5-minute blocks (instructions, preparation, speech task, and mental arithmetic task).

#### Exposure

**Fig 3:**
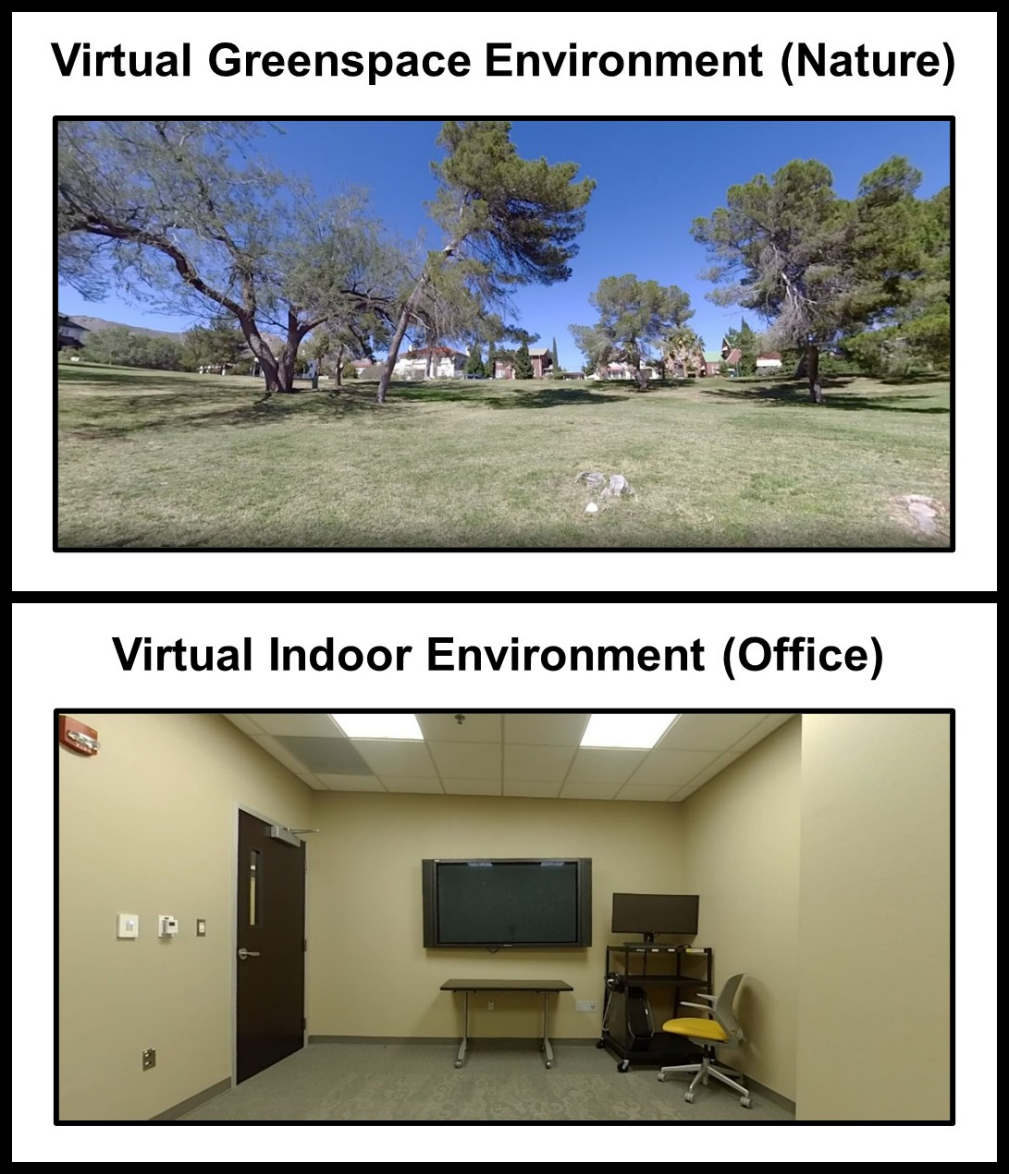
Virtual reality environments. Equirectangular depictions of the 360-degree 8k stationary recordings (Insta360 Pro™, Insta360.Inc, Shenzhen, Hong Kong) delivered in virtual reality using a commercial headset (VIVE Pro™, HTC Corporation, Taoyuan, Taiwan) after participants experienced an acute stressor. The virtual greenspace environment (nature) was recorded at a public park within the same county as the university where the exposure experiment was conducted. The virtual indoor environment (office) was recorded at the same university office used for the experiment. Participants were seated in a chair for the duration of the exposure (10 min).

Participants were then exposed to one of two randomly assigned immersive virtual environmental conditions consisting of nature (a local public park with distance houses) or an indoor office (the same office used for the experiment, but in virtual reality). Images of these environments are presented in Fig 3. A technician conducted the random assignment, blinding the experimenter to the condition group of the participant. The virtual exposures were delivered using a commercial headset (VIVE Pro™, HTC Corporation, Taoyuan, Taiwan) and the virtual environments consisted of 360° videos, recorded using an 8k 360° camera (Insta360 Pro™, Insta360.Inc, Shenzhen, Hong Kong). We selected a public park in the same county as the university as this was a natural landscape that was familiar and accessible to the participants, concordant with epidemiological evidence that observed the effects of nature in residential settings [1–4]. We avoided settings with humans or animals, tall grass or dense tree cover, substantial landscape features or significant inclines, and recorded the setting in mid-spring under direct sunlight. We also used the office environment as the reference condition during analyses as this was the physical location of the participants during the virtual exposures, ensuring that the participants in the office condition did not experience a change of setting. This was done to minimize the impact of this condition, avoiding confounders that could be introduced by exposing participants to a different setting than their current location while also accounting for any potential effects of wearing the headset. Participants were seated in a chair in the middle of the office for the duration of the exposure (10 min). The length of this exposure was expected to induce a measurable effect, based on other studies observing effects after shorter periods [45, 46, 85–87].

#### Recovery

Subsequently, participants remained in the office for 40 min while resting in a chair for the recovery period. The length of this period was selected to ensure data was collected for at least 70 min after the initiation of the stressor, which is sufficient time to capture stress recovery (heart rate variability) based on prior literature [84, 88]. After this recovery period, participants were taken back to the examination room and the electrodes were removed. Participants were then debriefed, including the disclosure of misinformation (related to the stressor), marking the end of the exposure experiment.

### Stress recovery

Stress recovery was assessed using heart rate variability to index changes in autonomic activation (sympathetic and parasympathetic) throughout the experiment. Data were collected using BIOPAC MP150 for Windows and AcqKnoweldge data acquisition software (Biopac Systems Inc., Santa Barbara, CA). Raw signals were filtered through a BIOPAC ECG100C bioamplifier with a sampling frequency of 1kHz and set to record heart rate (HR) from 40–180 beats per minute. Data were broken up into four segments corresponding to the study periods (baseline, stressor, exposure, and recovery; see Fig 2).

Time, frequency, and non-linear domain metrics were analyzed using Kubios software (3.4.3 [89]), using an automatic correction algorithm for artifacts [90]. Time domain analyses included the root mean square of successive RR interval differences (RMSSD) which reflects vagal-mediated changes in beat-to-beat variance [69]. Frequency analyses included low frequency (LF: 0.04 to 0.15 Hz) and high frequency (HF: 0.15 to 0.4 Hz) metrics to calculate the LF/HF ratio which reflects sympatho-vagal balance [69] and was expressed in power bands of normalized units. Non-linear analyses used return maps (plotting every interval against the prior interval) to estimate the width of the eclipse (SD1) which reflects vagal-mediated changes in beat-to-beat variance [69], and the length of the eclipse (SD2) which reflects sympatho-vagal balance [69]. Distinct sympathetic (SNS) and parasympathetic (PNS) tone indexes were then estimated as a composite of time domain (e.g., HR intervals, RR intervals) and non-linear domain (e.g., SD2, SD1) metrics [89]. While these indexes are relatively novel in research applications, evidence has demonstrated they provide highly reliable assessments of the corresponding branch of the autonomic nervous system [91–94]. Cleaned data were log-transformed to normalize model residuals and correct for skewed distributions. These metrics were then separated into two profiles to index higher sympathetic (LF/HF, SNS, HR↑) or parasympathetic activation (RMSSD, PNS, HR↓) across the study periods.

### Stress susceptibility

Susceptibility to stress was indicated by both distal (socioeconomic status and early life stress) and proximal (inflammatory reactivity and glucocorticoid resistance) indicators, as implicated by our framework (see Fig 1).

#### Distal indicators (socioeconomic status and early life stress)

Perceived socioeconomic status was measured using the MacArthur Scale of Subjective Social Status [95] which evaluates subjective social standing, reflecting impressions of current circumstances, background characteristics, and future opportunities. Scores are represented on a 10-point scale with higher scores representing higher perceived socioeconomic status.

Early life stress was measured using the Adverse Childhood Experience Questionnaire [96]. This questionnaire includes 10 self-report items on experiences of childhood abuse (emotional, physical, and sexual), neglect (emotional and physical), and household dysfunction (divorce, maternal violence, substance abuse, mental illness, and incarceration). Each endorsement of a childhood stressor is scored as one point with a total score representing the sum of these endorsements.

#### Proximal indicators (pro-inflammatory phenotype)

Bioassays of serum drawn before the baseline assessment were used to assess: (a) inflammatory reactivity, and (b) glucocorticoid resistance as proximal indicators of susceptibility to stress. Inflammatory reactivity was assessed by the quantity of IL-6 produced by monocytes from an in vitro exposure to lipopolysaccharide bacteria. Glucocorticoid resistance was assessed by the sensitivity of monocytes to dexamethasone; the quantity of dexamethasone needed to reduce 50% of IL-6 produced (IC_50_) from the same lipopolysaccharide exposure.

We used the protocol validated by Miller et al [33, 34, 97] to assess these indicators using monocyte-corrected values (correcting for the absolute number of monocytes in circulation to account for cellular disparities). Concentrations of IL-6 in supernatants were measured in duplicate using the MILLIPLEX MAP Human High Sensitivity Cytokine panel (catalog # HSCYTMAG-60SK) from MilliporeSigma Corp. (Burlington, MA USA); analyzed on a Luminex 200 analyzer running xPOTENT® (3.1) software (Luminex Corp., Austin, TX). IL-6 concentrations were reported as pg/mL (detection limit: 0.11). Controls were within the expected range (inter-assay CV: 6.17%). IC_50_ calculations were performed using GraphPad Prism software (9.1.1; San Diego, CA). Data were log-transformed to normalize model residuals and correct for skewed distributions. Larger values were indicative of higher inflammatory reactivity and glucocorticoid resistance in response to the in vitro bacterial challenge.

### Data analysis

First, we confirmed that the stressor induced cardiovascular arousal (increased sympathetic and reduced parasympathetic activation during the stressor period, relative to the baseline period) and that stress recovery was captured following the stressor (increased parasympathetic and reduced sympathetic activation during the exposure and recovery periods, relative to the stressor period) using linear mixed effect models with random intercepts (participants) and restricted maximum likelihood estimation to explore the main effect of time for each autonomic metric. This was accomplished using pairwise contrasts with corrections for multiple comparisons (Tukey’s HSD). Second, we tested if the nature condition was associated with better stress recovery relative to the office condition, also using mixed effect models, but with an interaction term (time x condition group) for each autonomic metric. In these mixed effect interaction models, the office condition was used as a reference for the pairwise contrasts.

Our hypothesis was tested using a series of multiple regression models, with one sequence per autonomic metric (outcome), to test the unique effect of each susceptibility indicator (socioeconomic status, early life stress, inflammatory reactivity, and glucocorticoid resistance), independently, conserving statistical power. These models used assessments during the recovery period, where the greatest differences between conditions were observed across all autonomic metrics. Each model included the corresponding assessment of the autonomic metric at baseline (to explore changes over time and account for individual differences), two main effects (condition group and susceptibility indicator), and an interaction term (condition group x susceptibility indicator) to test our hypothesis that the slope of the linear relationship between the susceptibility indicator and autonomic metric differed between the nature and office (reference) conditions. Susceptibility indicators were centered to aid in interpretations and all assumptions for multiple regression were confirmed before interpreting the models. We also tested the sensitivity of the effects within all multiple regression models using binary representations of the susceptibility indicators (delineated by the median). These sensitivity analyses were conducted to explore whether our results remained consistent across various representations of susceptibility (binary versus continuous).

Although we utilized alphas (0.05 level) to report significant findings, we focused our interpretations on forest plots (standardized coefficients and confidence intervals) that were used to infer directional trends and relative effect sizes across all regression models, regardless of statistical significance. Data were analyzed using R (3.6.3) with the “lmerTest” package for mixed effect models, the “emmeans” package for contrasts, the “effectsize” package for standardized coefficients, and the “ggplot2” package for generating plots.

## Results

No significant differences in participant demographics, health status, susceptibility indicators, or autonomic metrics at baseline were noted across the condition groups, as expected from randomization (Table 1). Bivariate correlations for the susceptibility indicators (S1 Table) and autonomic metrics (S2 Table) are provided in the supplemental materials.

**Table 1:** Participant characteristics.

Characteristic	M ± SD or N (%)			
	Total (*n* = 64)	Office (*n* = 32)	Nature (*n* = 32)	*p*
Demographics				
Age (Years)	22.70 ± 3.35	22.16 ± 3.21	23.25 ± 3.44	.19
Hispanic or Latino	53 (82.8%)	25 (78.1%)	28 (87.5%)	.51
White ^A^	55 (85.9%)	26 (81.3%)	29 (90.6%)	.47
Health Status				
BMI (kg/m2)	24.32 ± 3.27	24.50 ± 3.81	24.14 ± 2.67	.66
SBP (mmHg)	121.67 ± 10.24	122.56 ± 10.91	120.78 ± 9.62	.49
DBP (mmHg)	73.27 ± 7.69	72.88 ± 7.90	73.66 ± 7.59	.69
Pulse (per min)	61.73 ± 8.89	62.50 ± 9.34	60.97 ± 8.49	.50
Depressive Symptoms ^B^	3.53 ± 2.81	3.66 ± 2.62	3.41 ± 3.02	.72
Sleep Duration (Hours) ^C^	6.93 ± 0.83	7.00 ± 0.94	6.86 ± 0.70	.51
Sleep Quality ^D^	2.38 ± 0.75	2.53 ± 0.72	2.22 ± 0.75	.09
Physical Activity ^E^	22.32 ± 15.45	22.31 ± 18.26	22.33 ± 12.38	.99
Susceptibility				
Socioeconomic Status ^F^	4.00 ± 1.75	3.97 ± 1.75	4.03 ± 1.77	.89
Early Life Stress ^G^	1.56 ± 1.71	1.59 ± 1.97	1.53 ± 1.44	.89
Inflammatory Reactivity ^H^	1.17 ± 0.41	1.15 ± 0.43	1.19 ± 0.39	.68
Glucocorticoid Resistance ^H^	2.02 ± 0.19	2.02 ± 0.19	2.03 ± 0.19	.90
Autonomic Activation (Baseline)				
RMSSD (ms)	73.84 ± 45.66	69.98 ± 32.13	77.70 ± 56.34	.50
PNS (nu) ^I^	1.29 ± 1.59	1.19 ± 1.34	1.38 ± 1.82	.62
LF/HF (nu)	1.01 ± 0.86	0.90 ± 0.57	1.30 ± 1.08	.28
SNS (nu) ^I^	-0.74 ± 0.85	-0.72 ± 0.95	-0.76 ± 0.76	.86
HR (bpm)	61.04 ± 8.10	60.95 ± 8.35	61.13 ± 7.97	.93

P-values represent between-group differences using t-tests or chi-square tests for the corresponding characteristic. (A) Black: *n* = 5, Asian: *n* = 2, Native American: *n* = 1, Pacific Islander: *n* = 1. (B) Patient Health Questionnaire. (C) Past month average sleep duration; (D) Past month average sleep quality, from 1 “very good” to 5 “very bad”. (E) Past month days x hours of exercise. (F) MacArthur Scale of Subjective Social Status; (G) Adverse Childhood Experience Questionnaire; (H) In vitro bacterial challenge; (I) Composite of time and non-linear domain metrics.

The stressor induced observable cardiovascular arousal as indicated by significant differences in autonomic activation between the baseline and stressor periods, with higher arousal observed during the stressor period (increased sympathetic and reduced parasympathetic activation, relative to the baseline period; S1 Fig). Significant between-group differences were not observed during the baseline (*p* = .71 –.93) or stressor periods (*p* = .74 –.99), indicating similar stress reactivity across the condition groups.

We also observed clear indications of stress recovery following the stressor, indicated by significant differences between the stressor and subsequent periods, trending towards lower arousal over time (increased parasympathetic and reduced sympathetic activation, relative to the stressor period; S1 Fig). Significant between-group differences were also not observed during the exposure (*p* = .42 –.99) or recovery (*p* = .26 –.60) periods. However, non-significant trends were consistently in the direction of better stress recovery for participants in the nature versus office condition during both post-stressor periods (S2 Fig), with the largest differences observed during the recovery period.

### Modifying effects of susceptibility on stress recovery

**Fig 4:**
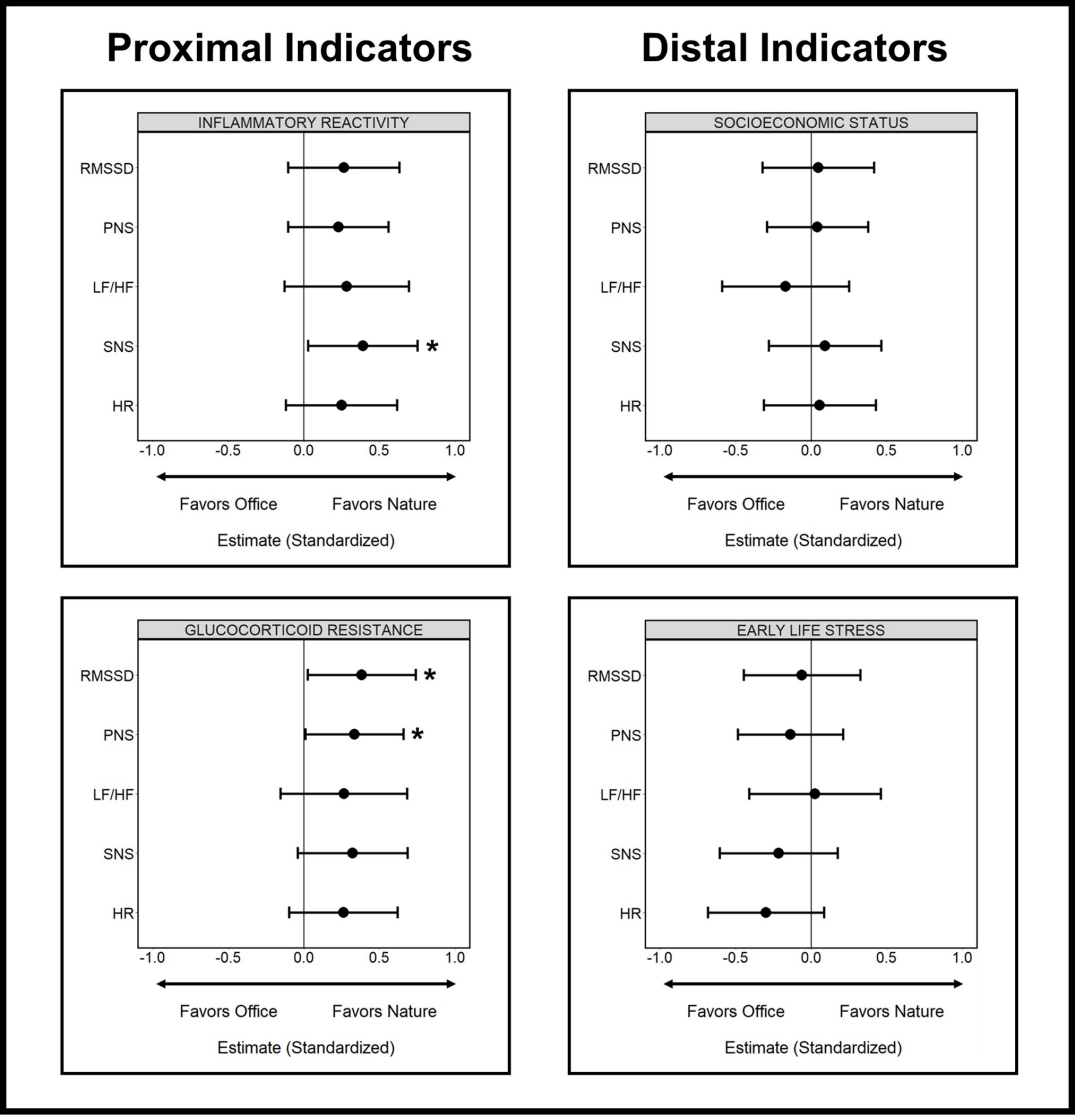
Forest plots of standardized effects (Continuous interaction terms). Forest plots visualizing interaction terms (standardized coefficients and confidence intervals [95%]) across all multiple regression models. Within these plots, all models were specified so that among participants with higher susceptibility (continuous), positive interaction terms indicate greater stress recovery (increased parasympathetic and reduced sympathetic activation) in the nature versus office condition while negative terms indicate greater stress recovery in the office versus nature condition. Interaction terms at zero indicate no differences in the association between susceptibility and stress recovery (autonomic activation) by condition group. * *p* < .05.

We then tested our interaction terms (condition groups x susceptibility indicator) and interpreted global trends across all models, regardless of statistical significance, using forest plots with standardized coefficients and confidence intervals (Fig 4). Modest trends were observed among the interaction effects during the recovery period for the pro-inflammatory indicators; higher inflammatory reactivity and glucocorticoid resistance consistently trended in the direction of better stress recovery (increased parasympathetic and reduced sympathetic activation) in the nature condition versus office condition across all models. However, these same trends were not observed for socioeconomic status or early life stress which showed no discernable trends among the interaction effects with the condition groups on stress recovery.

**Fig 5:**
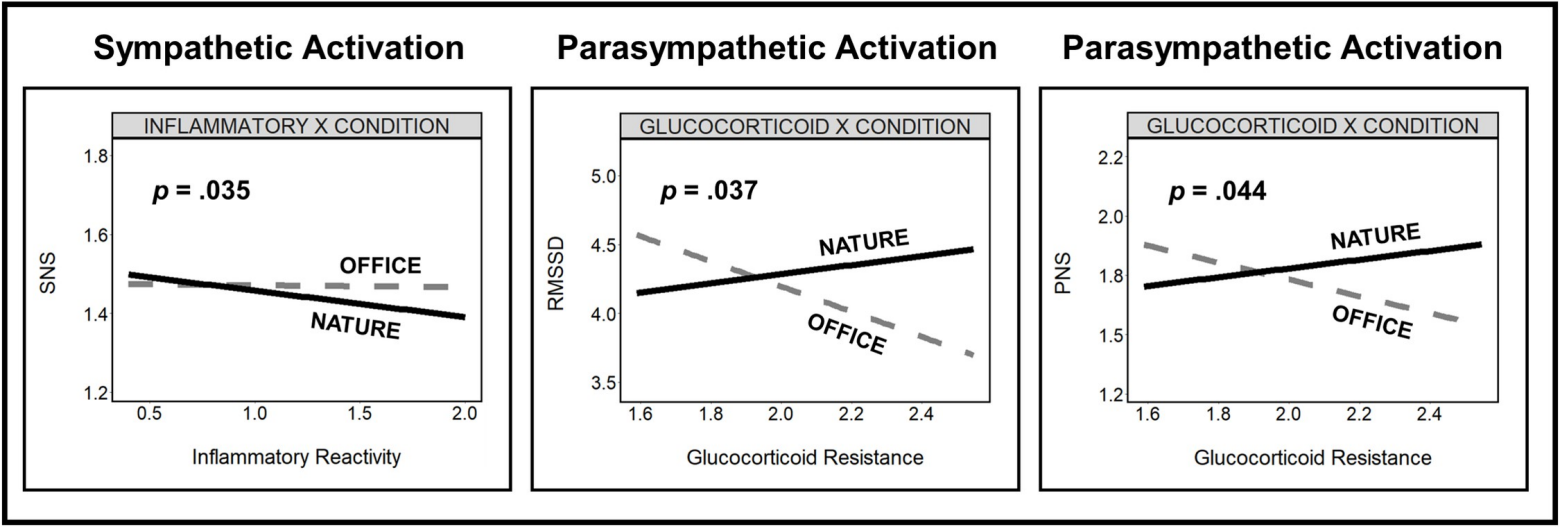
Interaction plots: Susceptibility by condition on autonomic activation (Recovery). Interaction plots visualizing significant associations (slopes) between susceptibility and stress recovery (autonomic activation) by condition group (nature [solid black line] versus office [dashed gray line]). Y-axes present sympathetic or parasympathetic activation using fitted values (unstandardized) from the corresponding regression model (baseline adjusted metric of autonomic activity [log] during the recovery period [40 min]). X-axes present susceptibility indicators using log-values. P-values (beta weights) represent the interaction term.

To better understand the characteristics of these trends, we then generated interaction plots for our significant interactions (Figs 5 and S3). Specifically, inflammatory reactivity was associated with greater reductions in sympathetic activation for participants in the nature versus office group during the recovery period (SNS: β = -0.390, 95% CI [-0.750, -0.029], *p* = .035), and glucocorticoid resistance was associated with greater increases in parasympathetic activation for participants in the nature versus office group during the recovery period (RMSSD: β = 0.382, 95% CI [0.024, 0.739], *p* = .037; PNS: β = 0.334 95% CI [0.009, 0.659], *p* = .044).

The direction of these significant interactions indicates the influence (slope) of a pro-inflammatory phenotype on autonomic activation differs between the environmental conditions. Specifically, a pro-inflammatory phenotype was associated with greater recovery (increased parasympathetic and reduced sympathetic activation) in the nature versus office environment, following sympathetic arousal induced by the stressor. The direction of the simple slopes for each condition suggests a pro-inflammatory phenotype had a positive trend on stress recovery in the nature condition (SNS: β = -0.335, 95% CI [-0.586, -0.083], *p* = .010; RMSSD: β = 0.134, 95% CI [-0.117, 0.385], *p* = .290; PNS: β = 0.186, 95% CI [-0.043, 0.414], *p* = 0.110) but a negative trend on stress recovery in the office condition (SNS: β = 0.055, 95% CI [-0.202, 0.311], *p* = .671; RMSSD: β = -0.248, 95% CI [-0.502, 0.007], *p* = .056; PNS: β = -0.148, 95% CI [-0.381, 0.084], *p* = .206), although these trends were modest and mostly non-significant.

### Sensitivity analyses

Sensitivity analyses using binary indicators suggested these trends were fairly consistent across continuous and binary representations of susceptibility. Specifically, these binary analyses again revealed no discernable trends for socioeconomic status or early life stress while inflammatory reactivity and glucocorticoid resistance consistently trended towards better stress recovery in the nature condition and worse recovery in the office condition (S4 Fig). It is also of note that binary trends for inflammatory reactivity and glucocorticoid resistance were larger and mostly significant relative to continuous trends (modest and mostly non-significant).

## Discussion

In this experimental study among healthy college-age males, we observed trend-level, partial support for our hypothesis that participants with higher susceptibility to stress would show greater recovery from acute stress when exposed to natural environments, compared to less susceptible participants. Specifically, we found that participants with a pro-inflammatory phenotype, indicated by higher inflammatory reactivity and glucocorticoid resistance, consistently trended towards better stress recovery (increased parasympathetic and reduced sympathetic activation) when exposed to simulated elements of natural environments after an acute psychosocial stressor. However, differences in stress recovery were not observed among participants in lower versus higher socioeconomic positions or among participants with higher versus lower exposure to stressors in early life.

While the conclusions that can be drawn from this evidence are tentative and limited to college-age males, overall our findings are supportive of the need for further research in larger and more diverse samples, given the fact that we observed expected trends for inflammatory reactivity and glucocorticoid resistance, following just a 10-minute virtual reality exposure, even among a sample of participants with limited variability in terms of socioeconomic status and early life stress. As the diverse body of evidence supporting our framework was derived from more representative samples, including both females and older adults, it is plausible these trends could be supported in larger and more generalizable studies that include participants from low socioeconomic backgrounds with higher exposure to stressors in early life. In the following sections, we discuss some potential implications if future research using broader samples aligns with our findings.

### Theoretical implications

These trend-level findings only supported specific elements of our framework (see Fig 1) where we expected that susceptible participants in lower versus higher socioeconomic positions, who were at risk for higher exposure to stressors in early life and a pro-inflammatory phenotype induced by early life stress, would exhibit greater cardiovascular benefits (better stress recovery) in natural settings. Specifically, our findings support the plausibility that participants with higher inflammatory reactivity and glucocorticoid resistance exhibit better stress recovery in natural settings. However, our findings did not support this plausibility for participants in lower versus higher socioeconomic positions, or among those with higher versus lower exposure to stressors in early life.

This was surprising, as these expectations were based on large-scale epidemiological and national population-based studies in Europe and North America, which have consistently shown that adults (males and females) living in socioeconomically deprived neighborhoods experience greater health benefits from the protective effects of residential nature exposure [1–4]. However, it is possible these associations were not replicated in this study due to our small convenience sample of male college students, and a relative lack of variability in our study population regarding socioeconomic status and early life stress. This might also explain why we did not observe associations between our indicators of socioeconomic status, early life stress, and a pro-inflammatory phenotype (S1 Table) as shown within experimental studies [27, 28, 30–35].

### Research implications

If future research in more diverse populations shows that adults with a pro-inflammatory phenotype experience greater health benefits in natural settings, this could carry substantial implications for public health research. Specifically, these findings could support an alternative conceptualization of susceptibility, predominantly used to explain the health risks associated with adverse environments, as increased environmental sensitivity, reflecting heightened responses to both adverse and protective environmental conditions.

These findings could also further support the evidence base for the Differential Susceptibility Hypothesis [59], which has centered on social conditions, by indicating that susceptible adults also experience greater health benefits in protective physical environments such as nature. This could provide opportunities for effective interventions through altering and improving physical environments with the intention of promoting positive well-being outcomes. This has been less of a focus in public health literature, which has predominantly centered on the experiences of susceptible individuals in adverse environments. Future research could also underscore the importance of reconsidering how susceptibility is indexed in public health research, highlighting the advantages of using proximal indicators (i.e., biological correlates of susceptibility to stress). Traditional self-report measures of socioeconomic status and early life stress are mechanistically distal from health outcomes, restricting their utility as indicators of susceptibility due to the increased likelihood of confounding and the need to account for counteracting exposures (e.g., parental attachment, social support [98, 99]), that could be unknown or immeasurable [100, 101]. For instance, not all adults in low socioeconomic positions or adults with high exposure to stressors in early life develop susceptibility to their environment due to protective factors. These measures also do not consistently yield biological indicators of susceptibility, limiting applications in the discerning of causal pathways.

In our investigation, only the pro-inflammatory indicators trended towards better stress recovery among participants exposed to a natural setting following an acute stressor. We encourage further experimental work on these causal pathways to employ assessments of inflammatory reactivity and glucocorticoid resistance, potentially representing objective and sensitive indicators of susceptibility to stress compared to distal indicators such as socioeconomic status and early life stress [100, 101]. To this end, if we define “susceptibility” as an increased sensitivity to environmental factors, and “vulnerability” as higher exposure to environmental factors [102], then socioeconomic status and early life stress arguably serve as better indicators of “vulnerability” than “susceptibility”.

Future researchers are also encouraged to explore other proximal indicators of susceptibility to stress as implicated by a pro-inflammatory phenotype (e.g., cortisol reactivity, sympathetic reactivity, cardiovascular reactivity, amygdala reactivity [27, 28]). However, there are some discrepancies in the literature regarding how early life stress alters these systems. For instance, some reviews suggest early life stress induces cortisol hyper-reactivity [27] while other reviews suggest early life stress induces cortisol hypo-reactivity [19], with either augmentation leading to negative health outcomes during adulthood [103]. Distinguishing between experiences of childhood trauma versus adversities [104] might explain some of these discrepancies in the literature [58], as emerging systematic evidence indicates cortisol hypo-reactivity is associated with experiences of trauma while cortisol hyper-reactivity is associated with experiences of adversities [23]. Therefore, it is important that future investigators also consider how distinguishing between experiences of trauma versus adversities influences the effects of inflammatory reactivity and glucocorticoid resistance.

### Strengths and limitations

We provide trend-level experimental evidence supporting the possibility that susceptibility to stress (indicated by a pro-inflammatory phenotype) could induce greater cardiovascular benefits from nature exposure (stress recovery), among a specific sub-group of adults (male college students). Further, our results are supported by high levels of internal validity due to the blinded experimental design, successful induction of stress during the stressor, and successful capture of stress recovery during the post-stressor periods. We also used stringent inclusion/exclusion criteria [76], advanced algorithms to clean the heart rate variability data [90], strategies to mitigate confounding factors (e.g., novelty effect of virtual reality [81], timing of the exposure experiment for circadian effects [82]), along with analyzing blood samples in duplicate using a validated protocol [33, 34, 97].

We also used a virtual paradigm to isolate sensory effects (e.g., visual, auditory [65]) and mitigate environmental confounding factors (e.g., noise, temperature, exercise [52]) that might have been encountered in real world settings. However, while virtual and real world exposures are comparable [65, 66] effect sizes are often attenuated in virtual paradigms [105], suggesting our effect sizes might have been larger had we used a real world paradigm. Nonetheless, the use of a virtual paradigm also provides implications for simulated nature-based interventions that might be remarkably simple but effective, such as windows with nature views [106] or indoor plants [107, 108].

However, the generalizability of our study was substantially limited, warranting caution when interpreting our results from a small sample (*n* = 64) of male college students who were primarily Hispanic/Latinx and lived in West Texas. Although we expected our sample to have a distribution of socioeconomic position, we observed limited variability in our measures of socioeconomic status and early life stress, potentially contributing to the null results associated with these variables. Consequently, our results may have been underpowered and future research is needed to explore these trends in larger and more diverse samples, including females and older adults, with more variability in terms of socioeconomic status, early life stress, current life stress, and protective factors.

The use of a current measure of socioeconomic status represents another limitation as evidence indicates that higher socioeconomic status during adulthood is unable to reverse the lifelong effects of early life stress [32, 35]. To this end, it is also possible our outcomes could have been influenced by other pathways attributed to childhood socioeconomic status that are independent of early life stress. Further, the measure we used could have oversimplified our assessments and it might have been more informative to use a composite index across a broader range of indicators (e.g., household income, maternal education) to account for the various pathways to which socioeconomic status could influence susceptibility to stress, beyond the psychological aspects of subjective social status. Despite these shortcomings, some evidence indicates subjective assessments provide stronger and more consistent associations with stress-related factors, compared to objective scales using earned income, educational attainment, and occupational status [95] which were remarkably homogeneous in our sample of college student, most of whom were in their teens or early twenties. However, future researchers should include more comprehensive measures of different pathways (objective and subjective) in early life.

Using adverse childhood experiences (specific childhood stressors [96]) as a proxy for early life stress represents another limitation, as this measure does not account for the perceived severity, intensity, duration, or frequency of these events [100], protective factors [101], or broad social contexts emphasized by the social determinates of health [109]. Future researchers should account for these factors with more comprehensive assessments. Regarding the experimental design, it is also possible the virtual office setting was not a perfect neutral reference as this was the same room where the participants experienced the stressor. However, a perfect neutral environmental condition might be unattainable, and we considered that a change from the same real to virtual office setting would be a good approximation of neutral change compared to a new environment. Further, another benefit of using the same real and virtual office setting was that it accounted for any potential effects of wearing the headset. However, future researchers should also explore these trends across different comparator environments.

Another concern with our approach is the multiple comparisons problem, as alpha inflation increases the risk of false positives when testing a large volume of models without corrections [110]. To address this challenge, we focused our interpretations on directional trends and relative effect sizes across all regression models (hypothesis tests), using forest plots with standardized coefficients and confidence intervals. Further, without pre-established cut-off ranges for our pro-inflammatory indicators, we used a median split relative to the distribution of our sample for the binary representations. There is also controversy in the literature regarding the LF metric, with some authors suggesting it reflects both sympathetic and parasympathetic activation [111]. Yet, evidence indicates this metric solely reflects sympathetic activation when it is expressed in normalized units [88, 112]. This is also complimented by our results, showing a tight alignment with another metric of sympathetic activation (SNS) from alternative domains (composite of time and non-linear metrics [89]). Despite these limitations, overall, our results were supportive of specific elements of our framework which builds upon evidence derived from larger and more diverse samples.

### Public health implications

Overall, our trend-level findings support the value of further investigation into the notion that a pro-inflammatory phenotype could be a potential mechanism explaining greater cardiovascular benefits of nature exposure among adults in lower versus higher socioeconomic positions. However, even if the differential effects of a pro-inflammatory phenotype are independent of socioeconomic status in more diverse samples, such findings could also hold substantial implications for public health.

Specifically, future findings might further support the notion that incorporating nature into urban settings could be a strategic intervention target to curb the prevalence of cardiovascular disease, as indicated by existing evidence [52–55], but especially among adults with a pro-inflammatory phenotype. Importantly, nature in urban settings is typically a safe, feasible, and cost-effective intervention target [113, 114] with potential as a complementary health approach [52]. For instance, urban nature: (a) could be installed as a passive intervention; (b) is a long-term intervention, fostering generational health; and (c) could be implemented through public health policy. Urban nature also provides multiple co-benefits, such as reducing exposure to air pollution [52], representing another cardiovascular benefit of nature exposure among adults with a pro-inflammatory phenotype. For instance, work by Olvera-Alvarez et al (including work in progress) indicates these adults might also experience stronger cardiovascular risks from air pollution [102]. Other helpful nature-based interventions could even be provided using windows with nature views [106] or indoor plants [107, 108].

Future findings could also support a reconceptualization of susceptibility in public health, shifting focus to sensitivity across all environmental conditions (adverse and protective). This is underscored by our findings, providing modest support that higher inflammatory reactivity and glucocorticoid resistance might not just be risk factors [27, 28] but also resources in protective environments, promoting cardiovascular benefits among male college students. While this concept is contained within the Differential Susceptibility Hypothesis [59], we provide trend-level evidence these effects might also pertain to protective physical environments, providing another avenue of integration with public health.

We also indicated susceptibility across a range of proximal and distal indicators with our results affording: (a) higher confidence for inflammatory reactivity and glucocorticoid resistance as indicators of susceptibility (sensitivity) to environmental factors (see Miller & Chen [33]; Muscatell et al [62]); (b) lower confidence for traditional self-report measures of socioeconomic status and early life stress (see Anda et al [100]; McEwen & Gregerson [101]). Further validation of these proximal indicators in larger and more diverse samples could significantly enhance assessments of susceptibility to environmental factors in research and clinical settings, promoting prevention efforts for public health strategies centered on reducing the prevalence of cardiovascular disease.

Ultimately, future research in line with this conceptual framework could lead to a greater understanding of the ways in which nature-based interventions could be leveraged to mitigate cardiovascular health disparities among susceptible groups. Specifically, future research could further underscore the value of integrating protective environments, such as nature, into public health strategies, especially for adults with a pro-inflammatory phenotype, who are particularly susceptible to health risks but also might stand to experience the greatest health benefits from protective environments. Through the use of proximal indicators, we can also more effectively identify potential mechanisms facilitating cardiovascular risks and benefits among susceptible groups and tailor public health interventions, taking into account individual differences in environmental sensitivity to mitigate cardiovascular health disparities.

## Supporting information

S1 Table Bivariate correlations (Susceptibility indicators). Distal indicators of susceptibility included socioeconomic status (SES) and adverse childhood experiences (ACE) while proximal indicators included inflammatory reactivity (IL6) and glucocorticoid resistance (IC_50_).
(TIF)

S2 Table Bivariate correlations (Baseline autonomic metrics). Autonomic metrics were separated into two profiles to index higher parasympathetic (RMSSD, PNS, HR↓) or sympathetic activation (LF/HF, SNS, HR↑).
(TIF)

S1 Fig Cardiovascular arousal and recovery (Autonomic activation). Time plots visualizing changes in sympathetic (LF/HF, SNS, HR↑) or parasympathetic (RMSSD, PNS, HR↓) activation throughout the experimental protocol, relative to the baseline period. X-axes present the study periods (baseline [10 min], stressor [20 min], exposure [10 min], recovery [40 min]). Y-axes present mean differences from the baseline period (Δ; points) and corresponding confidence intervals (95%; error bars) obtained from the pairwise contrasts, using corrections for multiple comparisons (mixed effect models without interaction terms). Asterisks above the error bars represent significant differences from the baseline period; asterisks below these error bars represent significant differences from the stressor period. Gray dashed lines highlight the mean value for the baseline period. Gray dotted lines highlight the mean value for the stressor period. *p < .05; **p < .001.
(TIF)

S2 Fig Nature exposure and stress recovery (Autonomic activation). Mean and error plots visualizing group differences in sympathetic (LF/HF, SNS, HR↑) or parasympathetic (RMSSD, PNS, HR↓) activation during the exposure (left) or recovery (right) periods. Y-axis present mean differences between the nature versus office group obtained from the pairwise contrasts (mixed effect models with interaction terms). Points and confidence intervals (95% error bars) represent the nature condition compared to the office condition (dashed black line; y-intercept at zero).
(TIF)

S3 Fig Interaction plots: Maximum information. Interaction plots visualizing significant associations (slopes) between susceptibility and stress recovery (autonomic activation) by condition group (nature [solid black line] versus office [dashed gray line]). Y-axes present sympathetic or parasympathetic activation using fitted values (unstandardized) from the corresponding regression model (baseline adjusted metric of autonomic activity [log] during the recovery period [40 min]). X-axes present susceptibility indicators using log-values. P-values denote the simple slope for the nature (black) or office (gray) condition; error bands represent the standard error for each slope. Points denote participants in the nature (black triangle) or office (gray circle) condition. Type III effects represent the interaction term.
(TIF)

S4 Fig Forest plots of standardized effects (Binary interaction terms). Forest plots visualizing interaction terms (standardized coefficients and confidence intervals [95%]) across all multiple regression models. Within these plots, all models were specified so that among participants with high versus low susceptibility (binary; median-value), positive interaction terms indicate greater stress recovery (increased parasympathetic and reduced sympathetic activation) in the nature versus office condition while negative terms indicate greater stress recovery in the office versus nature condition. Interaction terms at zero indicate no differences in the association between susceptibility and stress recovery (autonomic activation) by condition group. * *p* < .05.
(TIF)

## References

[1] Mitchell R, Popham F. Effect of exposure to natural environment on health inequalities: an observational population study. Lancet. 2008;372(9650):1655–60. doi: 10.1016/S0140-6736(08)61689-X 18994663

[2] Mitchell RJ, Richardson EA, Shortt NK, Pearce JR. Neighborhood environments and socioeconomic inequalities in mental well-being. Am J Prev Med. 2015;49(1):80–4. doi: 10.1016/j.amepre.2015.01.017 25911270

[3] Brown SC, Perrino T, Lombard J, Wang K, Toro M, Rundek T, et al. Health disparities in the relationship of neighborhood greenness to mental health outcomes in 249,405 U.S. Medicare beneficiaries. Int J Environ Res Public Health. 2018;15(3):430. doi: 10.3390/ijerph15030430 29494513 PMC5876975

[4] Rigolon A, Browning MHEM, McAnirlin O, Yoon HV. Green space and health equity: a systematic review on the potential of green space to reduce health disparities. Int J Environ Res Public Health. 2021;18(5):2563. doi: 10.3390/ijerph18052563 33806546 PMC7967323

[5] Badland H, Pearce J. Liveable for whom? Prospects of urban liveability to address health inequities. Soc Sci Med. 2019;232:94–105. doi: 10.1016/j.socscimed.2019.05.001 31075753

[6] Craig JM, Prescott SL. Planning ahead: the mental health value of natural environments. Lancet Planet Health. 2017;1(4):e128–e129. doi: 10.1016/S2542-5196(17)30068-2 29851597

[7] Tost H, Reichert M, Braun U, Reinhard I, Peters R, Lautenbach S, et al. Neural correlates of individual differences in affective benefit of real-life urban green space exposure. Nat Neurosci. 2019;22(9):1389–93. doi: 10.1038/s41593-019-0451-y 31358990

[8] Astell-Burt T, Feng X. Does the potential benefit of neighbourhood green space for body mass index depend upon socioeconomic circumstances and local built and transport environments? A test of the ‘equigenesis’ hypothesis in Australia. J Transp Health. 2017;5:S40. doi: 10.1016/j.jth.2017.05.327

[9] Feng X, Astell-Burt T. Do greener areas promote more equitable child health? Health Place. 2017;46:267–73. doi: 10.1016/j.healthplace.2017.05.006 28666236

[10] Tomita A, Vandormael AM, Cuadros D, Di Minin E, Heikinheimo V, Tanser F, et al. Green environment and incident depression in South Africa: a geospatial analysis and mental health implications in a resource-limited setting. Lancet Planet Health. 2017;1(4):e152–e162. doi: 10.1016/S2542-5196(17)30063-3 28890948 PMC5589195

[11] Evans GW. The environment of childhood poverty. Am Psychol. 2004;59(2):77–92. doi: 10.1037/0003-066X.59.2.77 14992634

[12] Evans GW, Kim P. Multiple risk exposure as a potential explanatory mechanism for the socioeconomic status–health gradient. Ann N Y Acad Sci. 2010;1186(1):174–89. doi: 10.1111/j.1749-6632.2009.05336.x 20201873

[13] Evans GW, Kim P. Childhood poverty and young adults’ allostatic load: the mediating role of childhood cumulative risk exposure. Psychol Sci. 2012;23(9):979–83. doi: 10.1177/0956797612441218 22825357

[14] Evans GW, Kim P. Childhood poverty, chronic stress, self-regulation, and coping. Child Dev Perspect. 2013;7(1):43–8. doi: 10.1111/cdep.12013

[15] Giano Z, Wheeler DL, Hubach RD. The frequencies and disparities of adverse childhood experiences in the U.S. BMC Public Health. 2020;20(1):1327. doi: 10.1186/s12889-020-09411-z 32907569 PMC7488299

[16] Hertzman C, Boyce T. How experience gets under the skin to create gradients in developmental health. Annu Rev Public Health. 2010;31:329–47. doi: 10.1146/annurev.publhealth.012809.103538 20070189

[17] Hertzman C. Putting the concept of biological embedding in historical perspective. Proc Natl Acad Sci U S A. 2012;109 Suppl 2:17160–7. doi: 10.1073/pnas.1202203109 23045673 PMC3477385

[18] Aristizabal MJ, Anreiter I, Halldorsdottir T, Odgers CL, McDade TW, Goldenberg A, et al. Biological embedding of experience: a primer on epigenetics. Proc Natl Acad Sci U S A. 2020;117(38):23261–9. doi: 10.1073/pnas.1820838116 31624126 PMC7519272

[19] Danese A, McEwen BS. Adverse childhood experiences, allostasis, allostatic load, and age-related disease. Physiol Behav. 2012;106(1):29–39. doi: 10.1016/j.physbeh.2011.08.019 21888923

[20] Agorastos A, Pervanidou P, Chrousos GP, Baker DG. Developmental trajectories of early life stress and trauma: a narrative review on neurobiological aspects beyond stress system dysregulation. Front Psychiatry. 2019;10:118. doi: 10.3389/fpsyt.2019.00118 30914979 PMC6421311

[21] Fagundes CP, Glaser R, Kiecolt-Glaser JK. Stressful early life experiences and immune dysregulation across the lifespan. Brain Behav Immun. 2013;27(1):8–12. doi: 10.1016/j.bbi.2012.06.014 22771426 PMC3518756

[22] Smith KE, Pollak SD. Early life stress and development: potential mechanisms for adverse outcomes. J Neurodev Disord. 2020;12(1):34. doi: 10.1186/s11689-020-09337-y 33327939 PMC7745388

[23] Hosseini-Kamkar N, Lowe C, Morton JB. The differential calibration of the HPA axis as a function of trauma versus adversity: a systematic review and p-curve meta-analyses. Neurosci Biobehav Rev. 2021;127:54–135. doi: 10.1016/j.neubiorev.2021.04.006 33857580

[24] Maniam J, Antoniadis C, Morris MJ. Early-life stress, HPA axis adaptation, and mechanisms contributing to later health outcomes. Front Endocrinol (Lausanne). 2014;5:73. doi: 10.3389/fendo.2014.00073 24860550 PMC4026717

[25] Manyema M, Norris SA, Richter LM. Stress begets stress: the association of adverse childhood experiences with psychological distress in the presence of adult life stress. BMC Public Health. 2018;18(1):835. doi: 10.1186/s12889-018-5767-0 29976168 PMC6034311

[26] Taylor SE. Mechanisms linking early life stress to adult health outcomes. Proc Natl Acad Sci U S A. 2010;107(19):8507–12. doi: 10.1073/pnas.1003890107 20442329 PMC2889360

[27] Miller GE, Chen E, Parker KJ. Psychological stress in childhood and susceptibility to the chronic diseases of aging: moving toward a model of behavioral and biological mechanisms. Psychol Bull. 2011;137(6):959–97. doi: 10.1037/a0024768 21787044 PMC3202072

[28] Nusslock R, Miller GE. Early-life adversity and physical and emotional health across the lifespan: a neuroimmune network hypothesis. Biol Psychiatry. 2016;80(1):23–32. doi: 10.1016/j.biopsych.2015.05.017 26166230 PMC4670279

[29] Godoy LD, Rossignoli MT, Delfino-Pereira P, Garcia-Cairasco N, de Lima Umeoka EH. A comprehensive overview on stress neurobiology: basic concepts and clinical implications. Front Behav Neurosci. 2018;12:127. doi: 10.3389/fnbeh.2018.00127 30034327 PMC6043787

[30] Carpenter LL, Gawuga CE, Tyrka AR, Lee JK, Anderson GM, Price LH. Association between plasma IL-6 response to acute stress and early-life adversity in healthy adults. Neuropsychopharmacology. 2010;35(13):2617–23. doi: 10.1038/npp.2010.159 20881945 PMC2978751

[31] Schreier HMC, Kuras YI, McInnis CM, Thoma MV, St Pierre DG, Hanlin L, et al. Childhood physical neglect is associated with exaggerated systemic and intracellular inflammatory responses to repeated psychosocial stress in adulthood. Front Psychiatry. 2020;11:504. doi: 10.3389/fpsyt.2020.00504 32581878 PMC7290130

[32] Miller GE, Chen E, Fok AK, Walker H, Lim A, Nicholls EF, et al. Low early-life social class leaves a biological residue manifested by decreased glucocorticoid and increased proinflammatory signaling. Proc Natl Acad Sci U S A. 2009;106(34):14716–21. doi: 10.1073/pnas.0902971106 19617551 PMC2732821

[33] Miller GE, Chen E. Harsh family climate in early life presages the emergence of a proinflammatory phenotype in adolescence. Psychol Sci. 2010;21(6):848–56. doi: 10.1177/0956797610370161 20431047 PMC3207635

[34] Ehrlich KB, Ross KM, Chen E, Miller GE. Testing the biological embedding hypothesis: is early life adversity associated with a later proinflammatory phenotype? Dev Psychopathol. 2016;28(4pt2):1273–83. doi: 10.1017/S0954579416000845 27691981 PMC5475361

[35] Miller G, Chen E. Unfavorable socioeconomic conditions in early life presage expression of proinflammatory phenotype in adolescence. Psychosom Med. 2007;69(5):402–9. doi: 10.1097/PSY.0b013e318068fcf9 17556642

[36] Appleton J. The experience of landscape. London: Wiley; 1975.

[37] Driver BL, Greene P. Man’s nature: innate determinants of response to natural environments. In: Children, nature, and the urban environment: proceedings of a symposium-fair. Upper Darby (PA): US Department of Agriculture, Forest Service, Northeastern Forest Experiment Station; 1977. pp. 62–70. Report No.: NE-30.

[38] Orians GH. Habitat selection: general theory and applications to human behavior. In: Lockard JS, editor. The evolution of human social behavior. Amsterdam: Elsevier; 1980. pp. 49–63.

[39] Ulrich RS. Aesthetic and affective response to natural environment. In: Altman I, Wohlwill JF, editors. Behavior and the natural environment. New York: Plenum Press; 1983. pp. 85–125.

[40] Hartig T, Mang M, Evans GW. Restorative effects of natural environment experiences. Environ Behav. 1991;23(1):3–26. doi: 10.1177/0013916591231001

[41] Ulrich RS, Simons RF, Losito BD, Fiorito E, Miles MA, Zelson M. Stress recovery during exposure to natural and urban environments. J Environ Psychol. 1991;11(3):201–30. doi: 10.1016/S0272-4944(05)80184-7

[42] Parsons R, Tassinary LG, Ulrich RS, Hebl MR, Grossman-Alexander M. The view from the road: implications for stress recovery and immunization. J Environ Psychol. 1998;18(2):113–40. doi: 10.1006/jevp.1998.0086

[43] Brown DK, Barton JL, Gladwell VF. Viewing nature scenes positively affects recovery of autonomic function following acute-mental stress. Environ Sci Technol. 2013;47(11):5562–9. doi: 10.1021/es305019p 23590163 PMC3699874

[44] Anderson AP, Mayer MD, Fellows AM, Cowan DR, Hegel MT, Buckey JC. Relaxation with immersive natural scenes presented using virtual reality. Aerosp Med Hum Perform. 2017;88(6):520–6. doi: 10.3357/AMHP.4747.2017 28539139

[45] Guo LN, Zhao RL, Ren AH, Niu LX, Zhang YL. Stress recovery of campus street trees as visual stimuli on graduate students in autumn. Int J Environ Res Public Health. 2019;17(1):148. doi: 10.3390/ijerph17010148 31878199 PMC6982156

[46] Yin J, Yuan J, Arfaei N, Catalano PJ, Allen JG, Spengler JD. Effects of biophilic indoor environment on stress and anxiety recovery: a between-subjects experiment in virtual reality. Environ Int. 2020;136:105427. doi: 10.1016/j.envint.2019.105427 31881421

[47] Bratman GN, Hamilton JP, Daily GC. The impacts of nature experience on human cognitive function and mental health. Ann N Y Acad Sci. 2012;1249(1):118–36. doi: 10.1111/j.1749-6632.2011.06400.x 22320203

[48] Bratman GN, Olvera-Alvarez HA, Gross JJ. The affective benefits of nature exposure. Soc Personal Psychol Compass. 2021;15(8):e12630. doi: 10.1111/spc3.12630

[49] Roe JJ, Ward Thompson C, Aspinall PA, Brewer MJ, Duff EI, Miller D, et al. Green space and stress: evidence from cortisol measures in deprived urban communities. Int J Environ Res Public Health. 2013;10(9):4086–103. doi: 10.3390/ijerph10094086 24002726 PMC3799530

[50] Ward Thompson C, Roe J, Aspinall P, Mitchell R, Clow A, Miller D. More green space is linked to less stress in deprived communities: evidence from salivary cortisol patterns. Landsc Urban Plan. 2012;105(3):221–9. doi: 10.1016/j.landurbplan.2011.12.015

[51] Ward Thompson C, Aspinall P, Roe J, Robertson L, Miller D. Mitigating stress and supporting health in deprived urban communities: the importance of green space and the social environment. Int J Environ Res Public Health. 2016;13(4):440. doi: 10.3390/ijerph13040440 27110803 PMC4847102

[52] Frumkin H, Bratman GN, Breslow SJ, Cochran B, Kahn PH Jr, Lawler JJ, et al. Nature contact and human health: a research agenda. Environ Health Perspect. 2017;125(7):075001. doi: 10.1289/EHP1663 28796634 PMC5744722

[53] Yeager RA, Smith TR, Bhatnagar A. Green environments and cardiovascular health. Trends Cardiovasc Med. 2020;30(4):241–6. doi: 10.1016/j.tcm.2019.06.005 31248691 PMC7995555

[54] Shanahan DF, Bush R, Gaston KJ, Lin BB, Dean J, Barber E, et al. Health benefits from nature experiences depend on dose. Sci Rep. 2016;6:28551. doi: 10.1038/srep28551 27334040 PMC4917833

[55] Mao G, Cao Y, Wang B, Wang S, Chen Z, Wang J, et al. The salutary influence of forest bathing on elderly patients with chronic heart failure. Int J Environ Res Public Health. 2017;14(4):368. doi: 10.3390/ijerph14040368 28362327 PMC5409569

[56] Belsky J, Bakermans-Kranenburg MJ, van Ijzendoorn MH. For better and for worse: differential susceptibility to environmental influences. Curr Dir Psychol. 2007;16(6):300–4. doi: 10.1111/j.1467-8721.2007.00525.x

[57] Boyce WT, Ellis BJ. Biological sensitivity to context: I. An evolutionary-developmental theory of the origins and functions of stress reactivity. Dev Psychopathol. 2005;17(2):271–301. doi: 10.1017/s0954579405050145 16761546

[58] Del Giudice M, Ellis BJ, Shirtcliff EA. The Adaptive Calibration Model of stress responsivity. Neurosci Biobehav Rev. 2011;35(7):1562–92. doi: 10.1016/j.neubiorev.2010.11.007 21145350 PMC3068241

[59] Ellis BJ, Boyce WT, Belsky J, Bakermans-Kranenburg MJ, van Ijzendoorn MH. Differential susceptibility to the environment: an evolutionary—neurodevelopmental theory. Dev Psychopathol. 2011;23(1):7–28. doi: 10.1017/S0954579410000611 21262036

[60] Belsky J, Pluess M. Beyond diathesis stress: differential susceptibility to environmental influences. Psychol Bull. 2009;135(6):885–908. doi: 10.1037/a0017376 19883141

[61] Albott CS, Forbes MK, Anker JJ. Association of childhood adversity with differential susceptibility of transdiagnostic psychopathology to environmental stress in adulthood. JAMA Netw Open. 2018;1(7):e185354. doi: 10.1001/jamanetworkopen.2018.5354 30646399 PMC6324405

[62] Muscatell KA, Moieni M, Inagaki TK, Dutcher JM, Jevtic I, Breen EC, et al. Exposure to an inflammatory challenge enhances neural sensitivity to negative and positive social feedback. Brain Behav Immun. 2016;57:21–9. doi: 10.1016/j.bbi.2016.03.022 27032568 PMC5011017

[63] American Psychological Association. APA dictionary of psychology: socioeconomic status (SES) [Internet]. American Psychological Association. 2023 [cited 2024 Jan 20]. Available from: https://dictionary.apa.org/socioeconomic-status.

[64] McLaughlin KA. Early life stress and psychopathology. In: Harkness K, Hayden EP, editors. The Oxford handbook of stress and mental health. Oxford: Oxford University Press; 2020. pp. 45–74.

[65] Browning MHEM, Mimnaugh KJ, van Riper CJ, Laurent HK, LaValle SM. Can simulated nature support mental health? Comparing short, single-doses of 360-degree nature videos in virtual reality with the outdoors. Front Psychol. 2020;10:2667. doi: 10.3389/fpsyg.2019.02667 32010003 PMC6974516

[66] Yin J, Zhu S, MacNaughton P, Allen JG, Spengler JD. Physiological and cognitive performance of exposure to biophilic indoor environment. Build Environ. 2018;132:255–62. doi: 10.1016/j.buildenv.2018.01.006

[67] Kim HG, Cheon EJ, Bai DS, Lee YH, Koo BH. Stress and heart rate variability: a meta-analysis and review of the literature. Psychiatry Investig. 2018;15(3):235–45. doi: 10.30773/pi.2017.08.17 29486547 PMC5900369

[68] Ernst G. Heart-rate variability-more than heart beats? Front Public Health. 2017;5:240. doi: 10.3389/fpubh.2017.00240 28955705 PMC5600971

[69] Shaffer F, Ginsberg JP. An overview of heart rate variability metrics and norms. Front Public Health. 2017;5:258. doi: 10.3389/fpubh.2017.00258 29034226 PMC5624990

[70] Boesch M, Sefidan S, Ehlert U, Annen H, Wyss T, Steptoe A, et al. Mood and autonomic responses to repeated exposure to the Trier Social Stress Test for Groups (TSST-G). Psychoneuroendocrinology. 2014;43:41–51. doi: 10.1016/j.psyneuen.2014.02.003 24703169

[71] Grassi G, Seravalle G, Mancia G. Sympathetic activation in cardiovascular disease: evidence, clinical impact and therapeutic implications. Eur J Clin Invest. 2015;45(12):1367–75. doi: 10.1111/eci.12553 26480300

[72] Hadaya J, Ardell JL. Autonomic modulation for cardiovascular disease. Front Physiol. 2020;11:617459. doi: 10.3389/fphys.2020.617459 33414727 PMC7783451

[73] van Bilsen M, Patel HC, Bauersachs J, Böhm M, Borggrefe M, Brutsaert D, et al. The autonomic nervous system as a therapeutic target in heart failure: a scientific position statement from the Translational Research Committee of the Heart Failure Association of the European Society of Cardiology. Eur J Heart Fail. 2017;19(11):1361–78. doi: 10.1002/ejhf.921 28949064

[74] Zhang DY, Anderson AS. The sympathetic nervous system and heart failure. Cardiol Clin. 2014;32(1):33–45. doi: 10.1016/j.ccl.2013.09.010 24286577 PMC5873965

[75] Norton BJ, Strube MJ. Understanding statistical power. J Orthop Sports Phys Ther. 2001;31(6):307–15. doi: 10.2519/jospt.2001.31.6.307 11411625

[76] Allen AP, Kennedy PJ, Dockray S, Cryan JF, Dinan TG, Clarke G. The Trier Social Stress Test: principles and practice. Neurobiol Stress. 2016;6:113–26. doi: 10.1016/j.ynstr.2016.11.001 28229114 PMC5314443

[77] Helminen EC, Morton ML, Wang Q, Felver JC. A meta-analysis of cortisol reactivity to the Trier Social Stress Test in virtual environments. Psychoneuroendocrinology. 2019;110:104437. doi: 10.1016/j.psyneuen.2019.104437 31536942

[78] Liu Q, Zhang W. Sex differences in stress reactivity to the Trier Social Stress Test in virtual reality. Psychol Res Behav Manag. 2020;13:859–69. doi: 10.2147/PRBM.S268039 33154681 PMC7605969

[79] Narvaez Linares NF, Charron V, Ouimet AJ, Labelle PR, Plamondon H. A systematic review of the Trier Social Stress Test methodology: issues in promoting study comparison and replicable research. Neurobiol Stress. 2020;13:100235. doi: 10.1016/j.ynstr.2020.100235 33344691 PMC7739033

[80] Kroenke K, Spitzer RL, Williams JB. The PHQ-9: validity of a brief depression severity measure. J Gen Intern Med. 2001;16(9):606–13. doi: 10.1046/j.1525-1497.2001.016009606.x 11556941 PMC1495268

[81] Chirico A, Gaggioli A. When virtual feels real: comparing emotional responses and presence in virtual and natural environments. Cyberpsychol Behav Soc Netw. 2019;22(3):220–6. doi: 10.1089/cyber.2018.0393 30730222

[82] Koch CE, Leinweber B, Drengberg BC, Blaum C, Oster H. Interaction between circadian rhythms and stress. Neurobiol Stress. 2016;6:57–67. doi: 10.1016/j.ynstr.2016.09.001 28229109 PMC5314421

[83] Kirschbaum C, Pirke KM, Hellhammer DH. The ’Trier Social Stress Test’—a tool for investigating psychobiological stress responses in a laboratory setting. Neuropsychobiology. 1993;28(1–2):76–81. doi: 10.1159/000119004 8255414

[84] Allen AP, Kennedy PJ, Cryan JF, Dinan TG, Clarke G. Biological and psychological markers of stress in humans: focus on the Trier Social Stress Test. Neurosci Biobehav Rev. 2014;38:94–124. doi: 10.1016/j.neubiorev.2013.11.005 24239854

[85] Hedblom M, Gunnarsson B, Iravani B, Knez I, Schaefer M, Thorsson P, et al. Reduction of physiological stress by urban green space in a multisensory virtual experiment. Sci rep. 2019;9:10113. doi: 10.1038/s41598-019-46099-7 31300656 PMC6625985

[86] Jiang B, Li D, Larsen L, Sullivan WC. A dose-response curve describing the relationship between urban tree cover density and self-reported stress recovery. Environ Behav. 2014;48(4):607–29. doi: 10.1177/0013916514552321

[87] Valtchanov D, Ellard C. Physiological and affective responses to immersion in virtual reality: effects of nature and urban settings. J Cyber Ther Rehabil. 2010;3(4):359–73.

[88] Lackschewitz H, Hüther G, Kröner-Herwig B. Physiological and psychological stress responses in adults with attention-deficit/hyperactivity disorder (ADHD). Psychoneuroendocrinology. 2008;33(5):612–24. doi: 10.1016/j.psyneuen.2008.01.016 18329819

[89] Tarvainen MP, Lipponen J, Niskanen JP, Ranta-Aho P. Kubios HRV Version 3 –user’s guide. Kuopio: University of Eastern Finland; 2017.

[90] Lipponen JA, Tarvainen MP. A robust algorithm for heart rate variability time series artefact correction using novel beat classification. J Med Eng Technol. 2019;43(3):173–81. doi: 10.1080/03091902.2019.1640306 31314618

[91] Cosmo C, Seligowski AV, Aiken EM, Van’t Wout-Frank M, Philip NS. Heart rate variability features as predictors of intermittent theta-burst stimulation response in posttraumatic stress disorder. Neuromodulation. 2022;25(4):588–95. doi: 10.1111/ner.13529 35670065 PMC8957628

[92] Lundell RV, Tuominen L, Ojanen T, Parkkola K, Räisänen-Sokolowski A. Diving responses in experienced rebreather divers: short-term heart rate variability in cold water diving. Front Physiol. 2021;12:649319. doi: 10.3389/fphys.2021.649319 33897457 PMC8058382

[93] Suminar DAA, Basri MI, Tammasse J, Bintang AK, Akbar M. Autonomic dysregulation in acute ischemic stroke patient with insomnia. Med Clín Práct. 2021;4 Suppl 1:100206. doi: 10.1016/j.mcpsp.2021.100206

[94] Sahoo TK, Mahapatra A, Ruban N. Stress index calculation and analysis based on heart rate variability of ECG signal with arrhythmia. In: 2019 innovations in power and advanced computing technologies (i-PACT); 2019 March 22–23; Vellore, India. New York: Institute of Electrical and Electronics Engineers; 2019. pp. 1–7.

[95] Adler NE, Epel ES, Castellazzo G, Ickovics JR. Relationship of subjective and objective social status with psychological and physiological functioning: preliminary data in healthy white women. Health Psychol. 2000;19(6):586–92. doi: 10.1037//0278-6133.19.6.586 11129362

[96] Felitti VJ, Anda RF, Nordenberg D, Williamson DF, Spitz AM, Edwards V, et al. Relationship of childhood abuse and household dysfunction to many of the leading causes of death in adults. The Adverse Childhood Experiences (ACE) Study. Am J Prev Med. 1998;14(4):245–58. doi: 10.1016/s0749-3797(98)00017-8 9635069

[97] Miller GE, Rohleder N, Stetler C, Kirschbaum C. Clinical depression and regulation of the inflammatory response during acute stress. Psychosom Med. 2005;67(5):679–87. doi: 10.1097/01.psy.0000174172.82428.ce 16204423

[98] Brinker J, Cheruvu VK. Social and emotional support as a protective factor against current depression among individuals with adverse childhood experiences. Prev Med Rep. 2016;5:127–33. doi: 10.1016/j.pmedr.2016.11.018 27981026 PMC5156603

[99] Crouch E, Radcliff E, Strompolis M, Srivastav A. Safe, stable, and nurtured: protective factors against poor physical and mental health outcomes following exposure to adverse childhood experiences (ACEs). J Child Adolesc Trauma. 2018;12(2):165–73. doi: 10.1007/s40653-018-0217-9 32318189 PMC7163854

[100] Anda RF, Porter LE, Brown DW. Inside the adverse childhood experience score: strengths, limitations, and misapplications. Am J Prev Med. 2020;59(2):293–95. doi: 10.1016/j.amepre.2020.01.009 32222260

[101] McEwen CA, Gregerson SF. A critical assessment of the adverse childhood experiences study at 20 years. Am J Prev Med. 2019;56:790–4. doi: 10.1016/j.amepre.2018.10.016 30803781

[102] Olvera-Alvarez HA, Kubzansky LD, Campen MJ, Slavich GM. Early life stress, air pollution, inflammation, and disease: an integrative review and immunologic model of social-environmental adversity and lifespan health. Neurosci Biobehav Rev. 2018;92:226–42. doi: 10.1016/j.neubiorev.2018.06.002 29874545 PMC6082389

[103] Turner AI, Smyth N, Hall SJ, Torres SJ, Hussein M, Jayasinghe SU, et al. Psychological stress reactivity and future health and disease outcomes: a systematic review of prospective evidence. Psychoneuroendocrinology. 2020;114:104599. doi: 10.1016/j.psyneuen.2020.104599 32045797

[104] Krupnik V. Trauma or adversity? Traumatology. 2019;25(4):256–61. doi: 10.1037/trm0000169

[105] Browning MHEM, Shipley N, McAnirlin O, Becker D, Yu CP, Hartig T, et al. An actual natural setting improves mood better than its virtual counterpart: a meta-analysis of experimental data. Front Psychol. 2020;11:2200. doi: 10.3389/fpsyg.2020.02200 33101104 PMC7554239

[106] Ulrich RS. View through a window may influence recovery from surgery. Science. 1984;224(4647):420–1. doi: 10.1126/science.6143402 6143402

[107] Park SH, Mattson RH. Effects of flowering and foliage plants in hospital rooms on patients recovering from abdominal surgery. Horttechnology. 2008;18(4):563–8. doi: 10.21273/HORTTECH.18.4.563

[108] Park SH, Mattson RH. Ornamental indoor plants in hospital rooms enhanced health outcomes of patients recovering from surgery. J Altern Complement Med. 2009;15(9):975–80. doi: 10.1089/acm.2009.0075 19715461

[109] National Academies of Sciences, Engineering, and Medicine; Health and Medicine Division; Board on Population Health and Public Health Practice; Committee on Community-Based Solutions to Promote Health Equity in the United States. Communities in action: pathways to health equity. Baciu A, Negussie Y, Geller A, Weinstein JN, editors. Washington (DC): US National Academies Press; 2017. 28418632

[110] Ranganathan P, Pramesh CS, Buyse M. Common pitfalls in statistical analysis: the perils of multiple testing. Perspect Clin Res. 2016;7(2):106–7. doi: 10.4103/2229-3485.179436 27141478 PMC4840791

[111] Akselrod S, Gordon D, Ubel FA, Shannon DC, Berger AC, Cohen RJ. Power spectrum analysis of heart rate fluctuation: a quantitative probe of beat-to-beat cardiovascular control. Science. 1981;213(4504):220–2. doi: 10.1126/science.6166045 6166045

[112] Task Force of the European Society of Cardiology and the North American Society of Pacing and Electrophysiology. Heart rate variability: standards of measurement, physiological interpretation and clinical use. Circulation. 1996;93(5):1043–65. 8598068

[113] Wolf KL, Measells MK, Grado SC, Robbins AST. Economic values of metro nature health benefits: a life course approach. Urban For Urban Green. 2015;14(3):694–701. doi: 10.1016/j.ufug.2015.06.009

[114] Brochu P, Jimenez MP, James P, Kinney PL, Lane K. Benefits of increasing greenness on all-cause mortality in the largest metropolitan areas of the United States within the past two decades. Front Public Health. 2022;10:841936. doi: 10.3389/fpubh.2022.841936 35619828 PMC9127575

