# Supplementary figures and images for: Susceptibility to stress and nature exposure: Unveiling differential susceptibility to physical environments; a randomized controlled trial
Source: PLoS One. 2024 Apr 17;19(4):e0301473. doi: 10.1371/journal.pone.0301473 (PMC11023441; doi:10.1371/journal.pone.0301473)

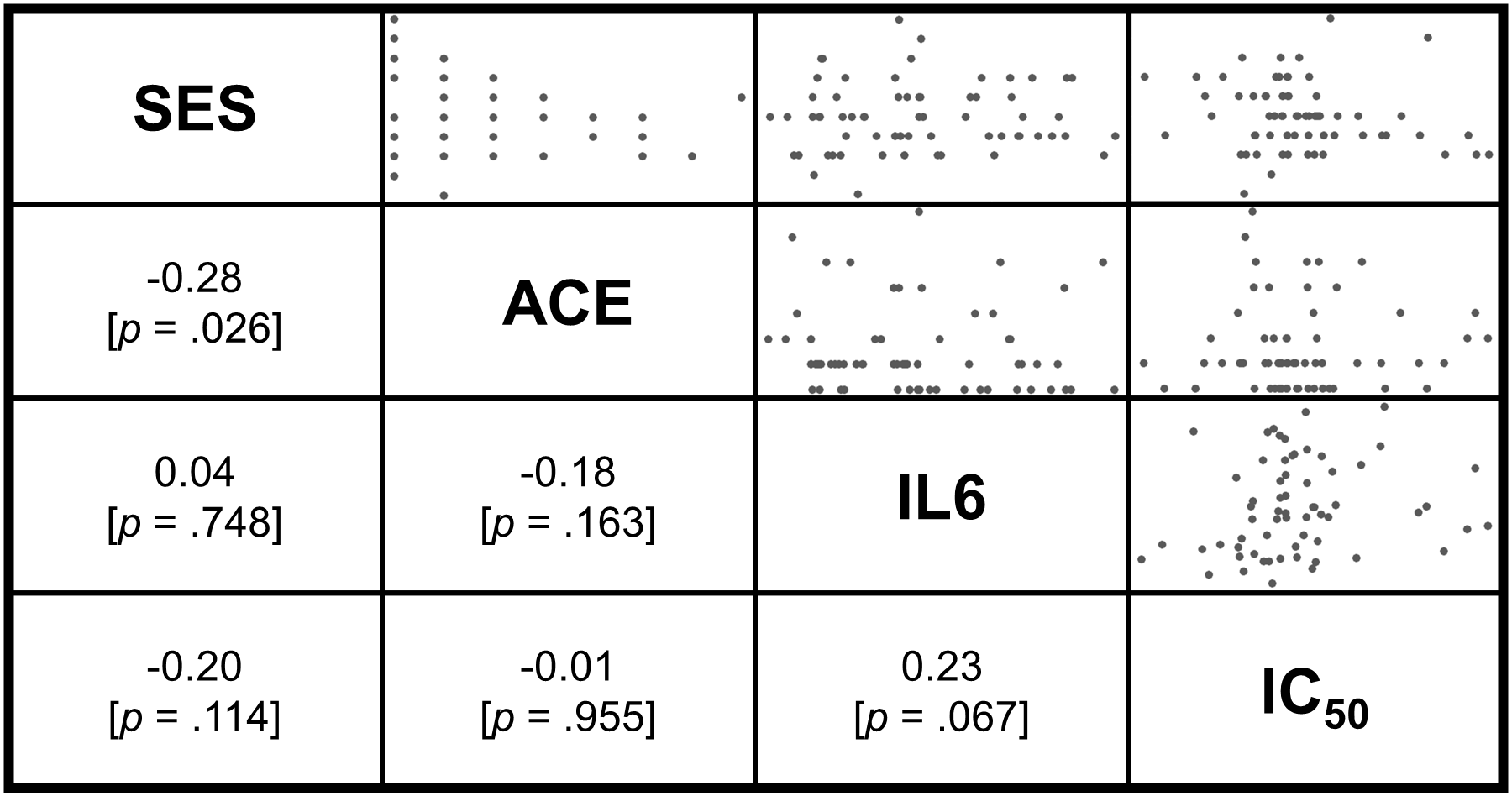

Supplement: S1 Table — Distal indicators of susceptibility included socioeconomic status (SES) and adverse childhood experiences (ACE) while proximal indicators included inflammatory reactivity (IL6) and glucocorticoid resistance (IC50). (TIF) [file pone.0301473.s001.tif]

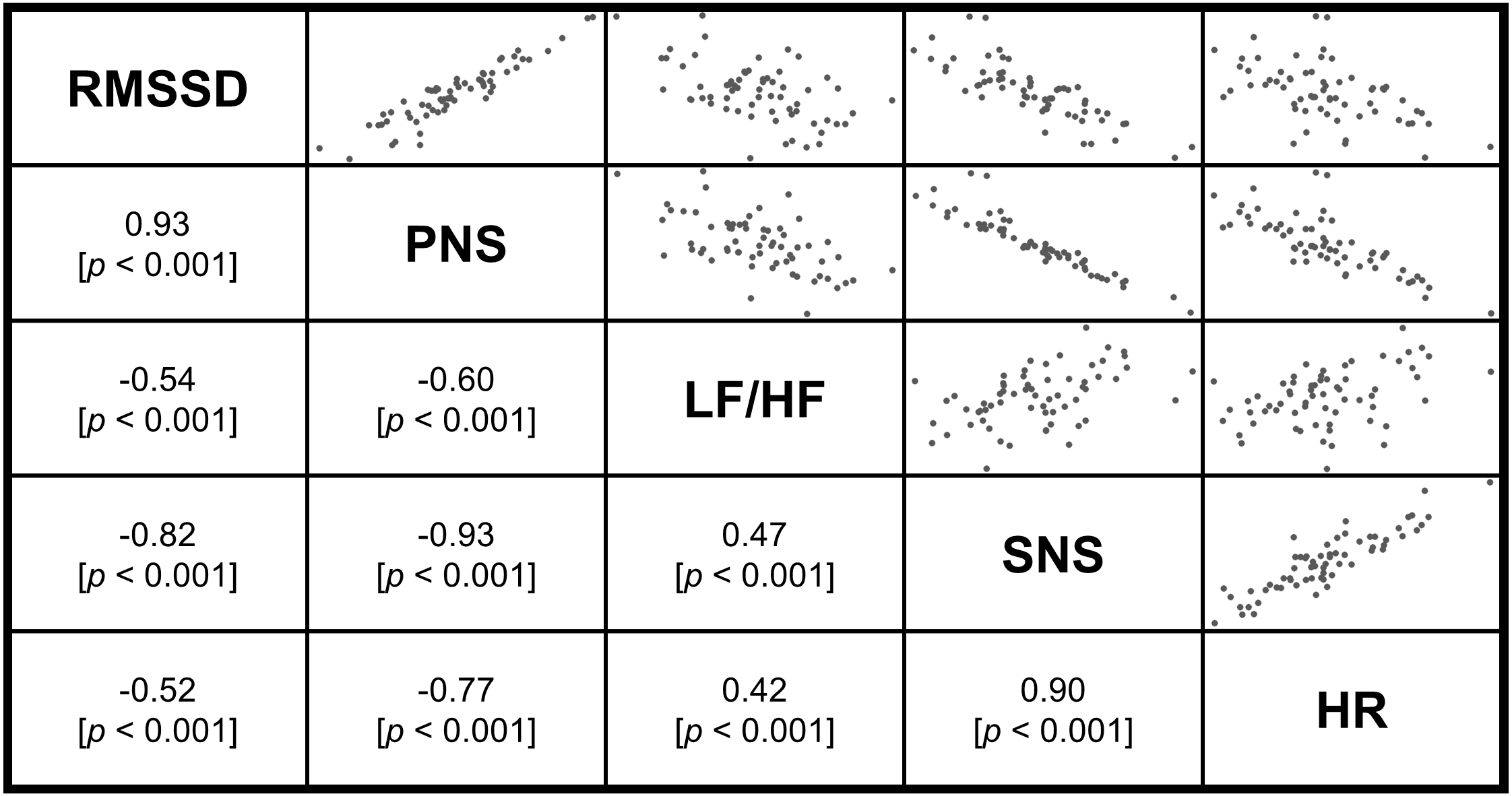

Supplement: S2 Table — Autonomic metrics were separated into two profiles to index higher parasympathetic (RMSSD, PNS, HR↓) or sympathetic activation (LF/HF, SNS, HR↑). (TIF) [file pone.0301473.s002.tif]

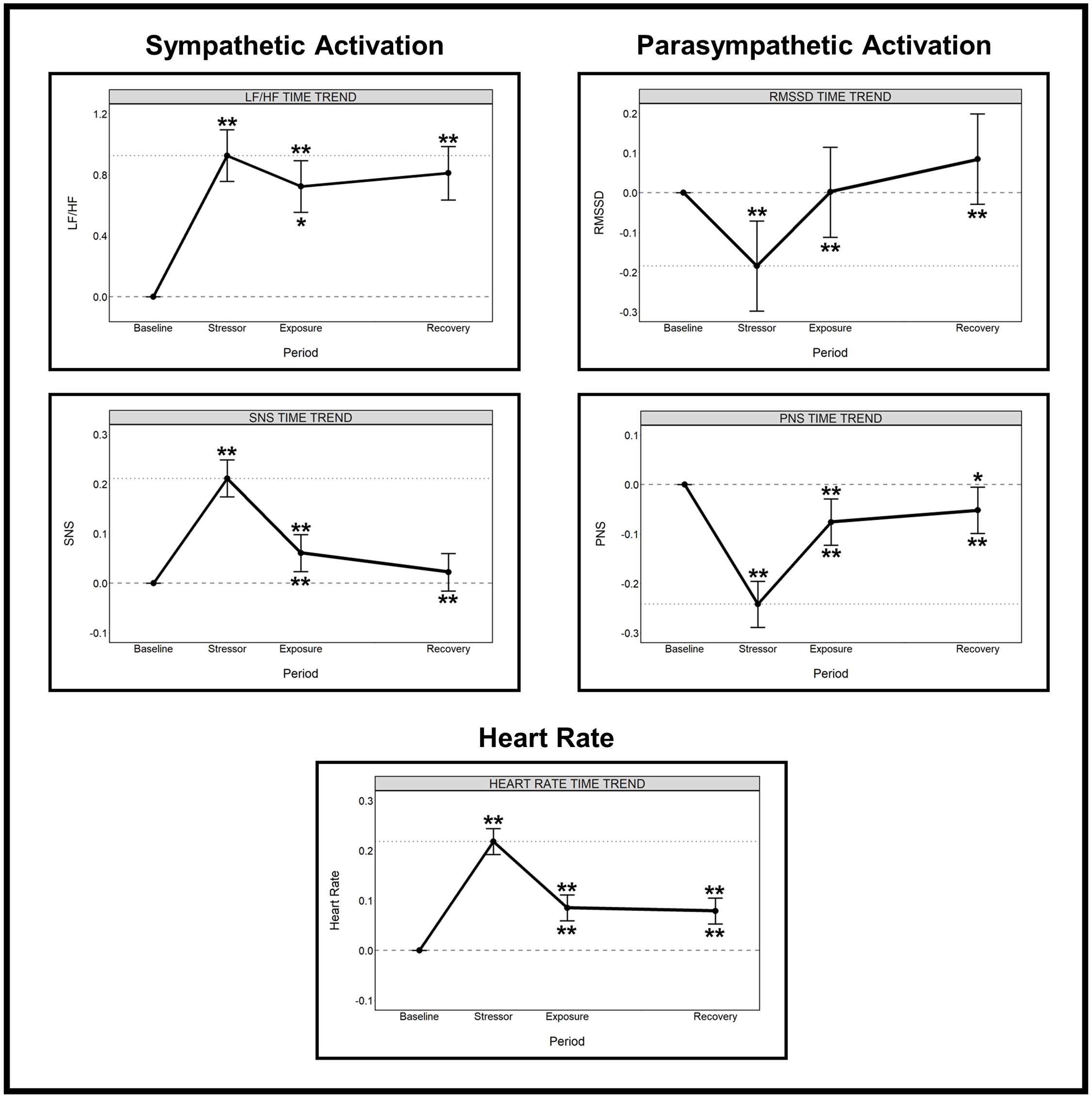

Supplement: S1 Fig — Time plots visualizing changes in sympathetic (LF/HF, SNS, HR↑) or parasympathetic (RMSSD, PNS, HR↓) activation throughout the experimental protocol, relative to the baseline period. X-axes present the study periods (baseline [10 min], stressor [20 min], exposure [10 min], recovery [40 min]). Y-axes present mean differences from the baseline period (Δ; points) and corresponding confidence intervals (95%; error bars) obtained from the pairwise contrasts, using corrections for multiple comparisons (mixed effect models without interaction terms). Asterisks above the error bars represent significant differences from the baseline period; asterisks below these error bars represent significant differences from the stressor period. Gray dashed lines highlight the mean value for the baseline period. Gray dotted lines highlight the mean value for the stressor period. *p < .05; **p < .001. (TIF) [file pone.0301473.s003.tif]

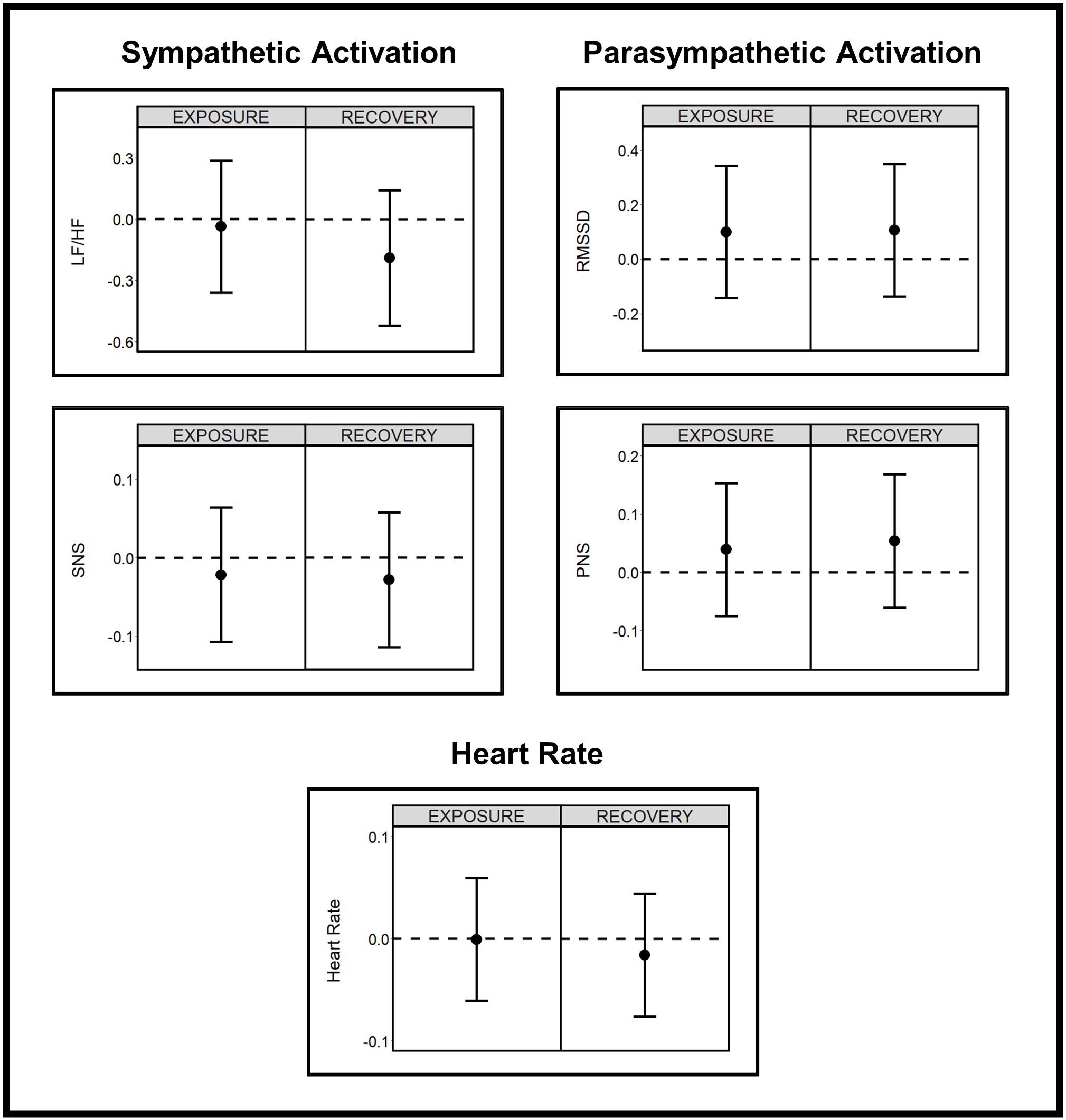

Supplement: S2 Fig — Mean and error plots visualizing group differences in sympathetic (LF/HF, SNS, HR↑) or parasympathetic (RMSSD, PNS, HR↓) activation during the exposure (left) or recovery (right) periods. Y-axis present mean differences between the nature versus office group obtained from the pairwise contrasts (mixed effect models with interaction terms). Points and confidence intervals (95% error bars) represent the nature condition compared to the office condition (dashed black line; y-intercept at zero). (TIF) [file pone.0301473.s004.tif]

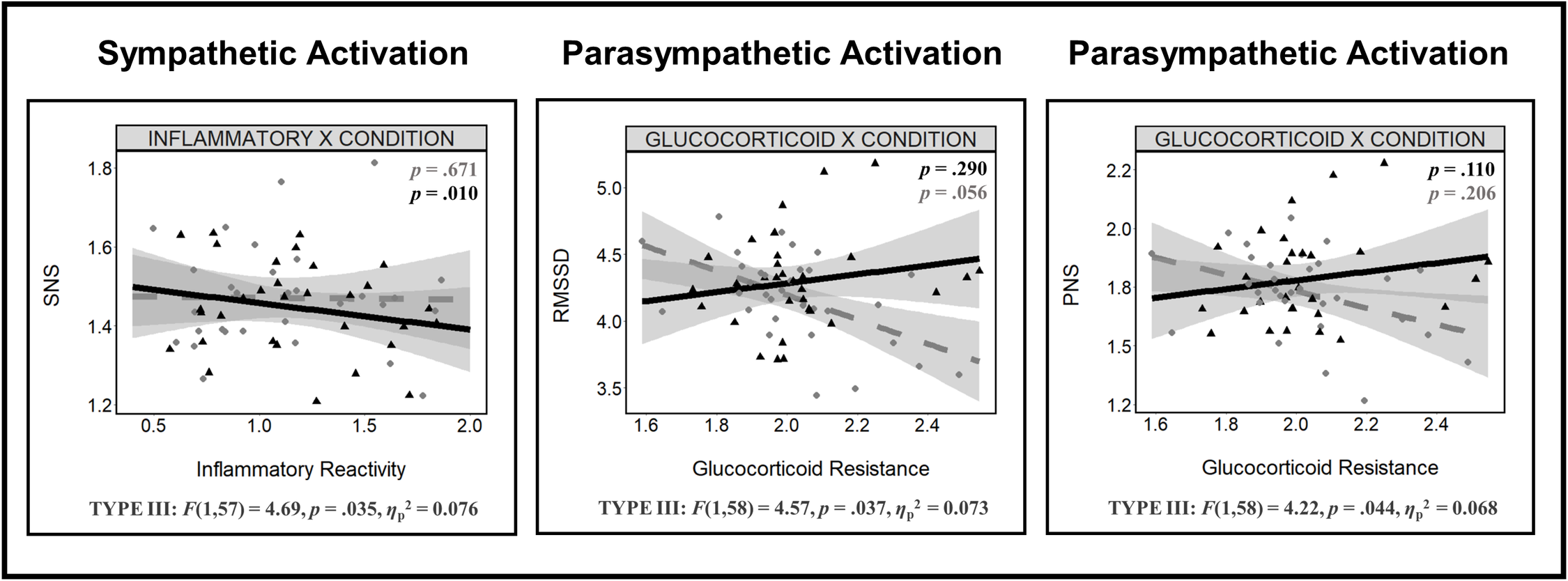

Supplement: S3 Fig — Interaction plots visualizing significant associations (slopes) between susceptibility and stress recovery (autonomic activation) by condition group (nature [solid black line] versus office [dashed gray line]). Y-axes present sympathetic or parasympathetic activation using fitted values (unstandardized) from the corresponding regression model (baseline adjusted metric of autonomic activity [log] during the recovery period [40 min]). X-axes present susceptibility indicators using log-values. P-values denote the simple slope for the nature (black) or office (gray) condition; error bands represent the standard error for each slope. Points denote participants in the nature (black triangle) or office (gray circle) condition. Type III effects represent the interaction term. (TIF) [file pone.0301473.s005.tif]

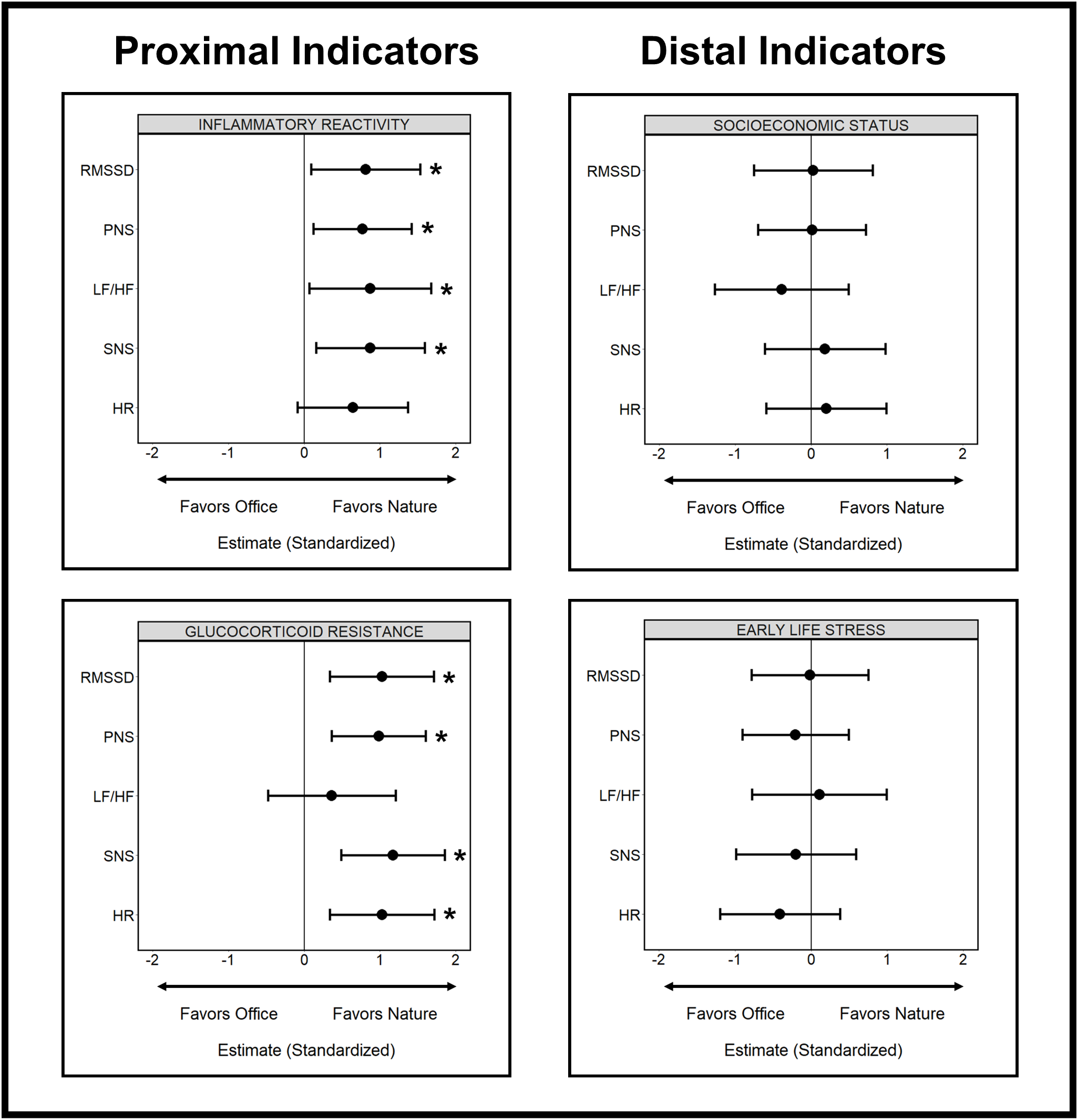

Supplement: S4 Fig — Forest plots visualizing interaction terms (standardized coefficients and confidence intervals [95%]) across all multiple regression models. Within these plots, all models were specified so that among participants with high versus low susceptibility (binary; median-value), positive interaction terms indicate greater stress recovery (increased parasympathetic and reduced sympathetic activation) in the nature versus office condition while negative terms indicate greater stress recovery in the office versus nature condition. Interaction terms at zero indicate no differences in the association between susceptibility and stress recovery (autonomic activation) by condition group. * p < .05. (TIF) [file pone.0301473.s006.tif]
